# The Effect of Health on the Elderly's Labor Supply in Rural China: Simultaneous Equation Models With Binary, Ordered, and Censored Variables

**DOI:** 10.3389/fpubh.2022.890374

**Published:** 2022-07-13

**Authors:** Na Tan, Liang Chang, Rui Guo, Baiyi Wu

**Affiliations:** ^1^Research Center for International Trade and Economics, Guangdong University of Foreign Studies, Guangzhou, China; ^2^School of Accounting, Guangdong University of Foreign Studies, Guangzhou, China; ^3^Research Center of Cross-Border M & As and Innovation Strategy, Guangdong University of Foreign Studies, Guangzhou, China; ^4^School of Finance, Guangdong University of Foreign Studies, Guangzhou, China

**Keywords:** The elderly's labor supply, hypertension, self-reported health, simultaneous equation models, binary Probit model, ordered Probit model, censored data

## Abstract

In this study, we examined the effect of health on the elderly's labor supply in rural China based on the data of the Chinese Health and Nutrition Survey (CHNS) from 1997 to 2006. We used simultaneous equations to address the endogeneity problem of health and estimate the models with censored data of labor supply by the full information maximum likelihood estimation. We found that the failing health does not significantly decrease the elderly's labor supply in rural areas when using both the subjective (self-reported health status) and objective (hypertension diagnosed or not) health indicators. Our finding indicates the phenomenon of “ceaseless toil” for the elderly in rural China, i.e., the elderly almost work their whole life even if they are not physically capable. The results remain robust when using a two-stage limited information maximum likelihood estimation.

## Introduction

With the decreasing fertility rate and rising life expectancy, the pace of population aging in China is much faster than that in the past (1). The aging problem may impose a heavy burden on society, and the easing of the burden mostly depends on when the elderly decide to retire (2). Although people eventually will be too sick to work, they may retire far before then. There are two possibilities of the retirement timing for the elderly, leading to different fiscal pressures. On the one hand, the elderly choose to retire and begin to receive pensions once they feel just slightly ill. This way of old-age care requires the government to allocate a great amount of fiscal budget to support the high level of social welfare. On the other hand, the elderly tend to work their whole life even if they are not physically capable to make a living as Benjamin et al. (3) found in China. Such a way of old-age care reduces the toil of the government but comes at the cost of reducing the welfare of the elderly. Therefore, it is a challenge for the government to find a balance between the welfare of the elderly and the growing pension burden when determining the retirement policy (4).

The key to addressing this policy challenge is to investigate how the elderly's labor supply will respond to their failing health. Therefore, this study examined the effect of health on the elderly's labor supply in rural China and investigated the retirement choice patterns of the elderly in rural China. We wanted the answer to whether the elderly in rural China retire in time when they feel just slightly ill, as the first pattern, or will work their whole life even if they are not physically capable, as the latter pattern. Holding other factors constant, if health has no significant effect on the elderly's labor, it means they follow the first pattern, but if there is a significantly negative effect, it is the second one.

Theoretically, the impact of health on the elderly's labor supply is uncertain. On the one hand, failing health will lead to lower labor productivity, which in turn results in unemployment (5). The less healthy a person is, the more likely he or she is to drop out of the labor market (6). On the other hand, poor health also has an income effect, which may increase the labor supply. Poorer health requires more medical care and health services (7). Therefore, the elderly need to provide more labor to afford the health services if they are limited in economic conditions, especially when the social security system is not well ensured. In this study, we attempted to test the theory that explains the elderly's labor supply with the data from rural China.

The data in China are suitable research sample to investigate the impact of health on the elderly's labor supply in developing countries. The economic conditions and retirement patterns differ remarkably in urban and rural China due to China's unique urban–rural dual social structure. The old-age security system in cities is relatively complete, while in rural areas, the system has not been fully established. In some poor families in rural areas, where the adult children cannot afford to support their parents, the older parents need to earn their own living by heavy farming work all their life, which leads to the phenomenon of “ceaseless toil” (3, 8). Therefore, the data on the elderly in rural China provide us with sufficient variations in the sample for estimation.

However, an empirical challenge lies in the measurement of health. In the current literature, scholars often use subjective and objective indicators to measure health. Subjective indicators include self-reported health status, self-reported days of disability, or limitations in activities. Objective health indicators are constructed based on medical tests of health conditions, such as height, weight, body mass index (BMI), grip strength, blood pressure, and limitations in activities of daily living (ADLs) (9–11). Nevertheless, both indicators have the endogeneity problem, which may cause biased estimation results when examining the effect of health on labor supply (12–14).

The endogeneity problem comes from several aspects: First, the reverse causation, which means that the health will affect people's labor supply, while the labor supply may also affect the health status. For example, the justification bias will lead to an endogeneity problem of reverse causation, which indicates that people may deliberately undervalue their health status for the excuse of their withdrawal from the labor market (15–17); second, the omitted variables, which means that health is often related to an individual's economic and social conditions, which also affect the labor supply behavior. If the factors of economic and social conditions are omitted in regressions, it will lead to biased estimation results. Compared to subjective health indicators, the objective health indicators do not have the endogeneity problem caused by justification bias but still have the reverse causality and omitted variable problems. Third, objective health indicators are more susceptible to measurement errors, which may lead to endogeneity as well (11). Since the measurements of objective health indicators mostly depend on medical instruments, people's emotions will sometimes be influenced by those instruments and then cause measurement errors.

In this study, we used simultaneous equations to solve the endogeneity problem in the health and labor supply model. The estimation of simultaneous equations not only reduces the difficulty of searching for proper instruments to address the endogeneity problem but also gets a more asymptotically efficient estimated result.

We contribute to the literature in three ways: First, this study adds to the literature on the causal effect of health on the elderly's labor supply and in particular to the few studies that examine the effect in developing countries (6, 18–20). The effect of health on the elderly's labor supply is controversial in the literature. Some studies find that health has a significant positive impact on labor supply, but some conclude that there is no such effect (3, 21–24). The reason for the controversy lies in the endogeneity problem of health variables and the measurements of labor supply. Therefore, in this study, we used the simultaneous-equation Tobit models with both the subjective and objective health indicators to achieve more robust and efficient estimated results. Our results help to resolve the controversy in supporting the conclusion of Benjamin et al. (3) that there is a phenomenon of “ceaseless toil” among the elderly in rural areas in China.

Second, to reveal the impact of health from different aspects, we used both the objective and subjective health indicators to measure the health level of the elderly and considered the objective indicator (hypertension diagnosed or not) as a binary variable and the subjective indicator (self-reported health status) as an ordered variable in estimation, which enriches the measures of health in literature. Using hypertension as a health indicator has several advantages. Hypertension can be accurately measured and has fewer measurement errors. In addition, hypertension is a common chronic disease in older adults, which is mostly diagnosed when people who are not yet retired face the decision of retirement, so there are sufficient variations in the sample of older people with different decisions (25–27).

Third, in this study, we first derived a logarithmic likelihood function of the joint distribution for the simultaneous equations including both a Tobit-type limited dependent variable and a binary choice (or an ordered) variable and then used the full information maximum likelihood (FIML) estimation method to obtain a more asymptotically efficient estimated result. There is no previous research deriving the likelihood function of simultaneous equations in this type and using FIML to estimate them, which can be used for further research of relevant models. Besides the better asymptotically efficiency, another advantage of the FIML estimation is that the correlation coefficient of residuals in labor and health equations can be directly estimated. Therefore, we can test the significance of the correlation coefficient directly to verify the existence of endogeneity and address the endogeneity by FIML estimation.

The rest of this study is organized as follows: Section “MATERIALS AND METHODS” introduces the data, variable modeling and estimation strategies. Section “RESULTS” shows the results, and section “DISCUSSION” discusses our results compared with other related findings. The final section presents the concluding points.

## Materials and Methods

### Data

We used the data from China Health and Nutrition Survey (CHNS) to investigate the effect of health on the labor supply of the elderly in rural China. Our sample includes individuals with rural household registration, comprising men aged 60 and above and women aged 55 and above. We gathered 3,535 observations from 1997, 2001, 2004, and 2006 with complete data on hypertension, self-reported health status, and working hours^1^.

For the age criteria of the sample, we used 60 years for men and 55 years for women, because the working-age population is defined as men aged 16–59 and women aged 16–54 as the benchmark in China^2^. The age criteria of the retirement policy for urban workers are the same as above, but there is no specific standard of retirement for many self-employed workers in rural areas. Therefore, we focused on the rural elderly who have reached the same age as urban workers and explored their health and labor hours.

Our sample from CHNS is representative of the elderly living in rural China for several reasons. First, the data from CHNS cover nine provinces (Guangxi, Guizhou, Heilongjiang, Henan, Hubei, Hunan, Jiangsu, Liaoning, and Shandong) in China, which vary greatly in economy and geography of rural areas. Second, the samples from CHNS were selected by a multistage and random cluster design. Selected counties were stratified into three different levels of income, and a weighted random sampling technique was used to choose four counties in each province. Finally, during our research period from 1991 to 2006, China experienced large-scale social and economic reforms; the living standards of Chinese rural families changed a lot and related questions were included in the questionnaires of CHNS. Thus, the dataset in CHNS could cover the most representative population in rural China.

### Variables

#### The Explained Variable: Labor Supply

From the data of CHNS, the annual working hours of the elderly in rural China are composed of three parts: the employed working hours, the self-employed agricultural working hours (including home gardening, collective and household farming, raising livestock and poultry, and collective and household fishing), and the self-employed non-agricultural working hours. We summed up these three parts of annual working hours to measure the variable of labor supply (*Laborhour*) and took it as the logarithmic form in regressions.

In our sample, about 31.5% (1,112/3,535 equals 31.5%) observations' annual working hours are zero, which means that, if some elderly quit the labor market after retirement, their labor supply will censor to zero, and the Tobit model addressing the censored data is more suitable for estimation than ordinary least square regressions. If we focus on the labor participation rate as a binary variable, it will underestimate the labor supply of the elderly. Therefore, we emphasized the detailed working hours of the elderly as censored data in estimation and used the Tobit model to reduce the estimation bias.

**Figure 1:**
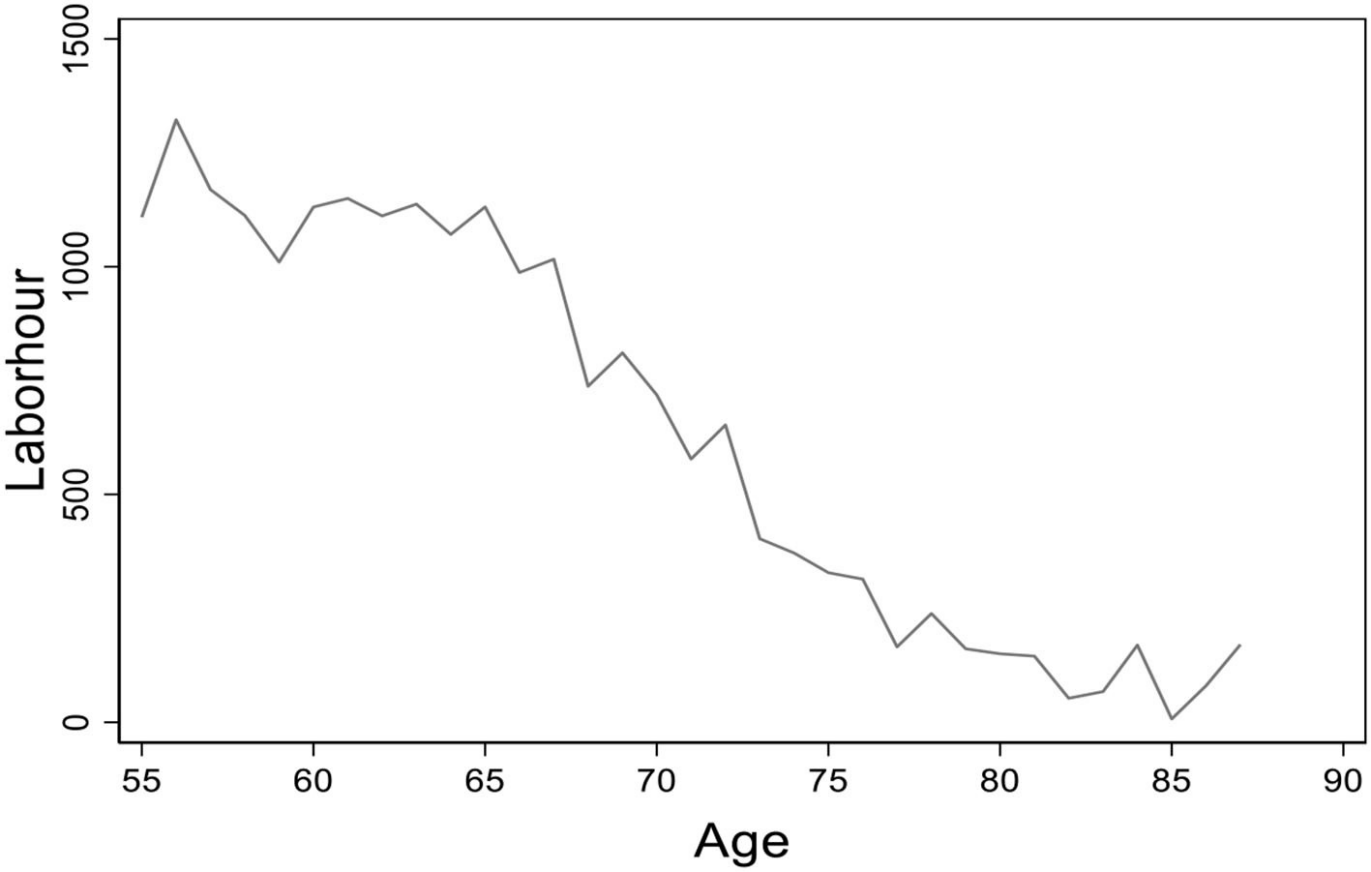
The age distribution of average working hours. Data from the China Health and Nutrition Survey (CHNS) database. The figure presents the age distribution of average working hours for the elderly in rural China.

Figure 1 shows the average working hours of the rural elderly in different age groups. The working hours decline along with the increase of age. Before the age of 70, the average working hours stay over 800, but there are almost no elderly who participate in the labor market after their 80s.

#### The Explanatory Variable: Health

In this study, we used both objective and subjective health indicators to investigate the effect of health on the elderly's labor supply from different perspectives. The objective indicator is hypertension diagnosed or not, and the subjective indicator is self-reported health status. Meanwhile, we emphasize the different data types of the indictors in estimation, i.e., hypertension as a binary variable and self-reported health as an ordered variable.

##### The Objective Indicator of Health: Hypertension

We used hypertension diagnosed or not (*Hypertension*) as an objective health indicator to measure health. The definition of the variable *Hypertension* is based on the benchmark of the World Health Organization (WHO) in 1999. If an individual's systolic blood pressure is not <140 mm Hg or diastolic blood pressure is not <90 mm Hg or “has been diagnosed as hypertension by the doctor,” we then define that the binary variable *Hypertension* equals to one but is otherwise zero. In the survey of CHNS, the doctor measured blood pressure three times, both systolic and diastolic, for each respondent. Since the first measurement is more likely to be affected by emotional fluctuations, we chose the average of the last two measurements to calculate the systolic and diastolic blood pressures.

Using hypertension to measure health has two advantages. One advantage is that hypertension can be accurately measured with fewer measurement errors and justification bias (25, 26). Another advantage is that hypertension is a common chronic disease in older Chinese, which provides sufficient variations in the sample (27, 28).

**Figure 2:**
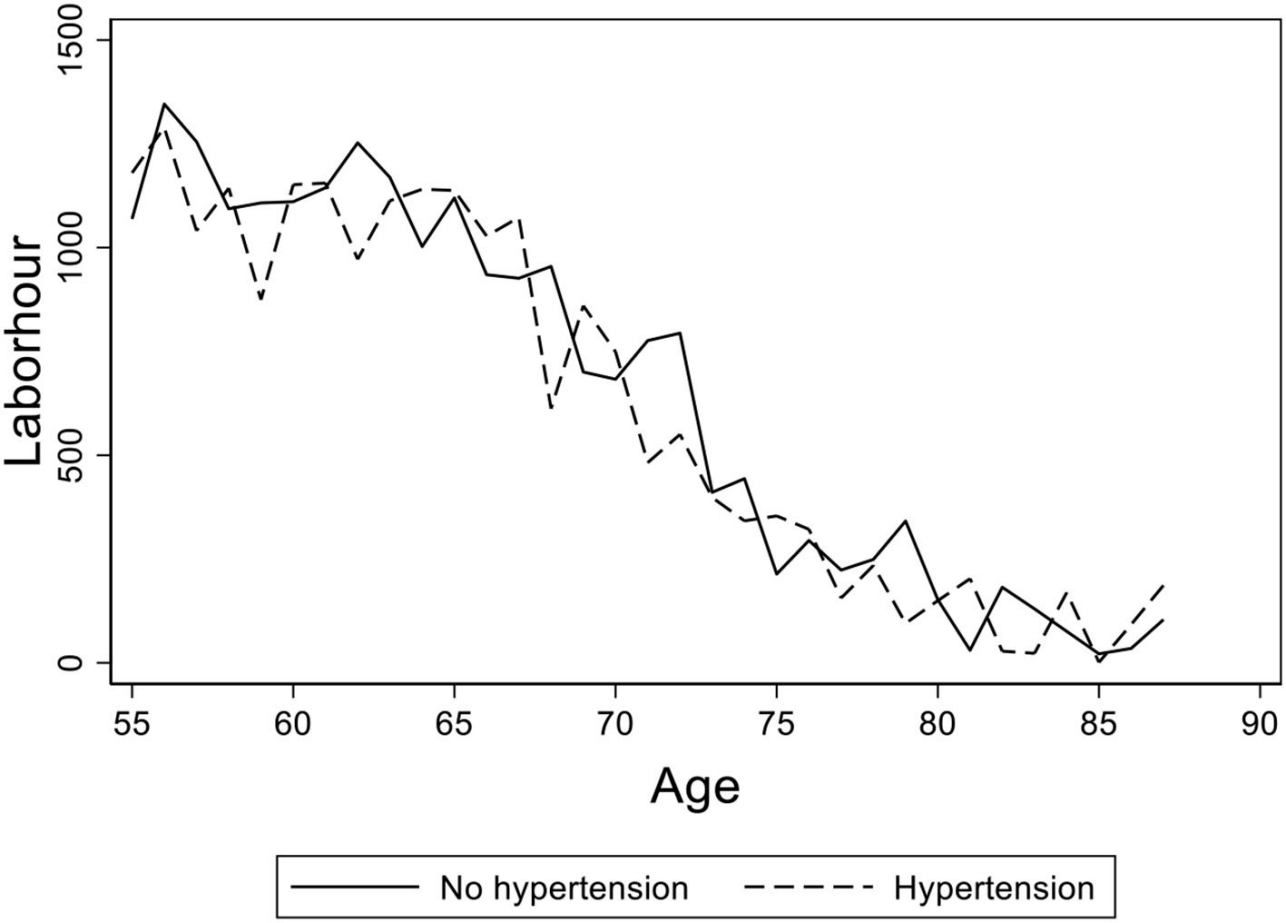
The age distribution of average working hours for the elderly with hypertension diagnosed or not. Data from the China Health and Nutrition Survey (CHNS) database. The figure shows the age distribution of average working hours for the elderly with hypertension diagnosed or not in rural China. The solid line shows the age path of the elderly without hypertension and the dashed line shows the ones with hypertension diagnosed.

Figure 2 displays the elderly's average working hours of different age groups in rural China with hypertension diagnosed or not. The solid line presents the working hours of the elderly without hypertension and the dashed line presents those of the elderly diagnosed with hypertension. We found that the average working hours of the two sub-samples are close, which implies that hypertension may not affect the elderly's working hours across different ages.

##### The Subjective Indicator of Health: Self-Reported Health

We also used the self-reported health status (*Selfhealth*) as a subjective health indicator to measure health. The CHNS questionnaire includes the question: “Right now, how would you describe your health compared to that of other people at your age?” We defined the variable *Selfhealth* as an ordered variable, which equals to one if the respondent chooses the answer “excellent” or “good,” equals to two if the choice is “fair,” and equals to three if the choice is “poor.”

**Figure 3:**
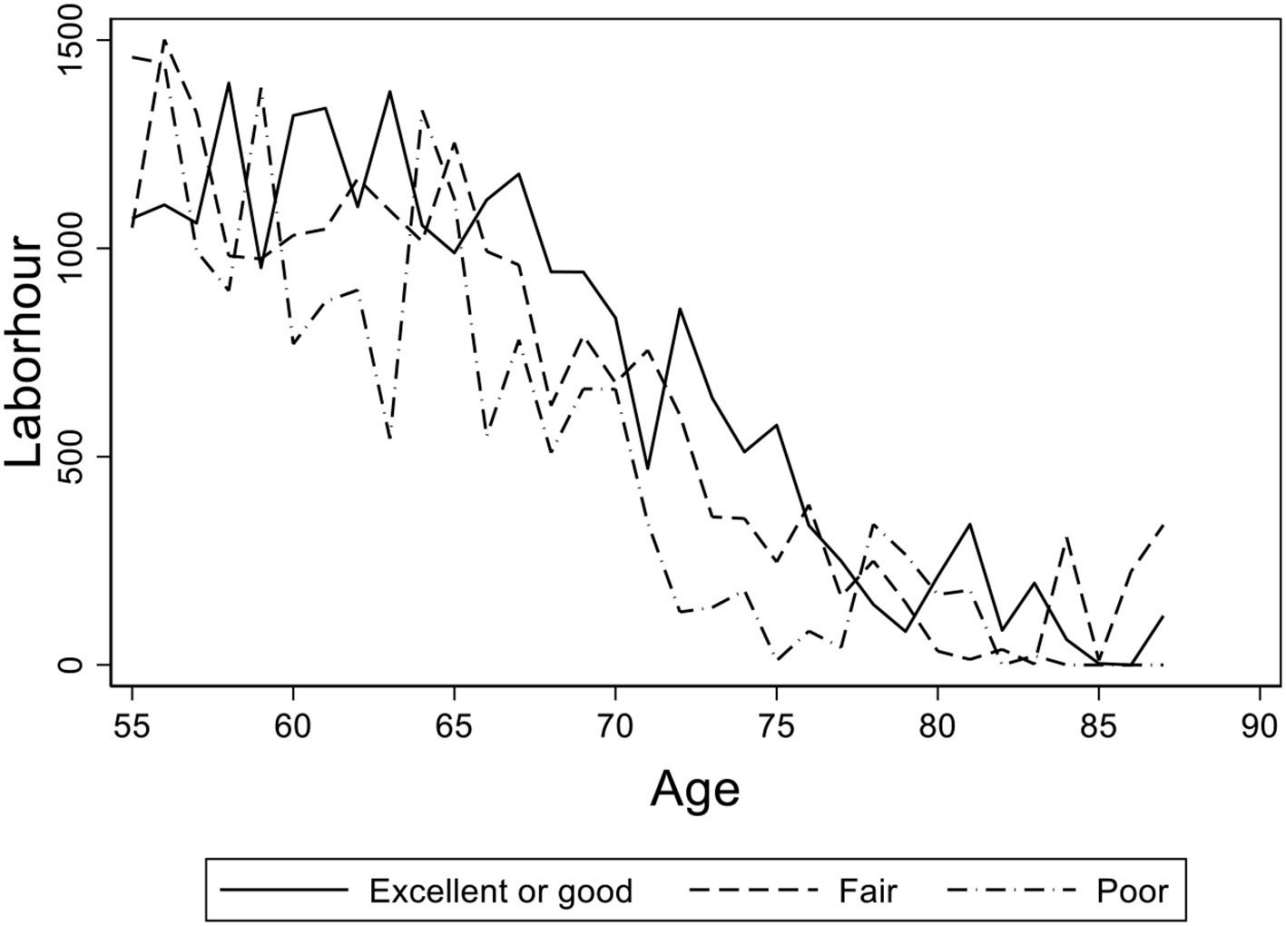
The age distribution of average working hours for the elderly with different self-reported health statuses. Data from the China Health and Nutrition Survey (CHNS) database. The figure shows the age distribution of average working hours for the elderly with different self-reported health statuses in rural China. The solid line shows the age path of the elderly with “excellent” or “good” health, the dashed line shows the ones with “fair” health, and the dash-dotted line shows the ones with “poor” self-reported health status.

In Figure 3, we present a graph of the elderly's average working hours of age groups with different self-reported health statuses. We found that the elderly's working hours are virtually the same between the groups of “good” and “fair,” and there is a little gap between “good” and “poor.” Figures 2, 3 provide preliminary descriptive evidence that the elderly in rural China may continue to work regardless of their failing health.

#### Other Control Variables

To examine the effect of health on the elderly's labor supply, the social and economic factors related to health and working hours are necessary to be controlled in regressions. Our control variables are from three aspects. First, for the individual characteristics, we controlled the age (*Age, Age2*), gender (*Male*), education levels (*Edu*), marital status (*Marr1, Marr2, Marr3*) of the elderly, and the year dummies. *Age2* is the square of an individual's age (*Age*) to control a non-linear effect in regression. *Male* is a dummy variable, which equals to one if the individual is a man but is otherwise zero. *Edu* is the total number of years of education, *Marr1* represents the marital status as divorced or separated, *Marr2* as widowed, and *Marr3* as married, respectively. We set the married individuals as the benchmark group in regressions.

Second, for the family factors, we controlled the family income (*Hhinc*) and wealth (*Hwealth*). *Hhinc* is defined as the annual household income and *Hwealth* is the accumulated family wealth, which were adjusted by CPI in 2009 and in the logarithmic form in regressions.

Third, according to the model settings of simultaneous equations, it is necessary to identify at least one variable only included in the health equation but not in the labor supply equation, which means that the identifying variables should affect health but not affect an individual's labor supply from other channels besides health. In this study, we used three variables, the amount of salt intake per person per meal (*Salt*), the square of the salt intake per person per meal (*Salt2*), and the alcohol consumption frequency (*Alcohol_fre*) as the identifying variables. Dahl (29) and MacMahon (30) indicated that high intake of salt and long-term alcohol consumption are positively correlated with hypertension.

Specifically, about the measurements, the nutrition survey of CHNS records the 3-day total actual salt consumption of the household, the 3-day total number of meals eaten at home per person, and the 3-day total number of persons who ate at home. Therefore, we divided the first value by the last two values to obtain the average amount of salt consumption per person per meal (*Salt*). In addition, the adult survey of CHNS records the frequency of alcohol consumption. We define the values of the variable *Alcohol_fre* as follows: 0 for never, 1 for not more than one time a month, 2 for one time or two times a month, 3 for once or twice a week, 4 for three to four times a week, and 5 for almost every day.

### Descriptive Statistics

The definitions and descriptive statistics of variables are shown in Table 1. The number of years of schooling is small, with an average of only 2.7 years, and about one-third of the elderly did not receive any formal education in our sample. The mean value of the annual working hours of the sample is about 869.4 h, which is relatively small because more than half of the elderly in the sample do not work. The zero values indicate that the explained variable is censored, so the results obtained by ordinary least squares will be biased, and the Tobit model is more suitable to address the censored data.

**Table 1:** Descriptive statistics.

**Variable**	**Definition**					
*Laborhour*	The annual working hours of each older person					
*Hypertension*	Dummy variable. Equals to 1 if the individual is diagnosed with hypertension, otherwise is 0					
*Self-health*	Ordered variable. Equals to 1 if the individual's self-reported health is “excellent” or “good”, 2 for the “fair”, and 3 for the “poor”					
*Age*	The age of each older person					
*Male*	Dummy variable. Equals to 1 if the individual is a man and 0 for women					
*Edu*	Number of years of schooling					
*Marr1*	Dummy variable. Equals to 1 if the marital status is divorced or separated, otherwise is 0					
*Marr2*	Dummy variable. Equals to 1 if the marital status is widowed, otherwise is 0					
*Marr3*	Dummy variable. Equals to 1 if the marital status is married, otherwise is 0					
*Hhinc*	The annual household income adjusted by CPI in 2009					
*Hwealth*	The accumulated household wealth adjusted by CPI in 2009					
*Drink_fre*	The alcohol consumption frequency. Equals to 0 if the individual never drinks, 1 for not more than one time a month, 2 for once or twice a month,					
	3 for one time or two times a week, 4 for three to four times a week, and 5 for almost every day					
*Salt*	The average amount of salt consumption per person per meal					
**Variable**	**Observations**	**Mean value**	**Standard deviation**	**Minimum**	**Median**	**Maximum**
*Laborhour*	3,535	869.437	1,175.701	0	390	5,824
*Hypertension*	3,535	0.566	0.496	0	1	1
*Self-health*	3,535	1.76	0.684	1	2	3
*Age*	3,535	66.008	7.545	55	65	87
*Male*	3,535	0.386	0.487	0	0	1
*Edu*	3,535	2.718	3.244	0	1	12
*Marr1*	3,535	0.029	0.167	0	0	1
*Marr2*	3,535	0.23	0.421	0	0	1
*Marr3*	3,535	0.728	0.445	0	1	1
*Hhinc_cpi*	3,535	9.068	1.146	5.055	9.177	11.17
*Hwealth*	3,535	0.419	0.972	0	0.114	6.308
*Drink_fre*	3,535	0.523	1.121	0	0	5
*Salt*	3,535	5.28	2.712	1.389	4.63	17.778

*Descriptive statistics of the sample, including 3,535 individuals with rural household registration in China from 1997, 2001, 2004, and 2006. The sample contains men aged 60 and above and women aged 55 and above*.

Further, we compare the mean values of the variables grouped by different health levels (hypertension diagnosed or not) in Table 2 and report the *t*-test results. The average annual working hours of rural elderly of the two groups are 343.3 and 278.1 h, respectively. Table 2 shows that there is a significant yet small difference between these two groups, which may be different from the data seen in Figure 2. Therefore, we need to investigate the effect of health on labor supply in regression by holding other confounding factors fixed as well as addressing the endogeneity problem of health.

**Table 2:** *T*-test for the differences between the elderly with hypertension diagnosed or not.

	**(1)**	**(2)**	**(3)**
	**No hypertension**	**Hypertension**	**Diff. (1)–(2)**
*Laborhour*	343.323	278.109	65.214***
*Age*	64.075	67.492	−3.417***
*Male*	0.345	0.416	−0.071***
*Edu*	2.915	2.567	0.348***
*Marr1*	0.027	0.030	−0.004
*Marr2*	0.198	0.255	−0.057***
*Marr3*	0.764	0.700	0.064***
*Hhinc_cpi*	9.154	9.003	0.151***
*Hwealth*	0.444	0.400	0.044
*Drink_fre*	0.511	0.532	−0.021
*Salt*	5.104	5.415	−0.31***

*The differences between the elderly with hypertension diagnosed or not in the sample. The variables are introduced in Table 1.*

***Indicate statistical significance at the 10, 5, and 1% levels, respectively.

Compared with those who are not diagnosed with hypertension, the elderly with hypertension are averagely older and poorer and there are more men than women. The frequency of alcohol consumption does not differ significantly between the hypertension and non-hypertension samples. However, the salt consumption is significantly higher in the hypertension group at the 1% level, which suggests that salt consumption is a proper identifying variable.

In Table 3, we compare the mean values of variables grouped by different self-reported health statuses as “excellent” or “good,” “fair,” and “poor,” and the results are consistent with Table 2. The elderly in rural areas evaluated their own health to be worse as they became older, and the women with lower household income and less education evaluated their own health status as significantly worse. In particular, the higher the frequency of drinking, the better the self-reported health is. The higher the salt consumption, the lower the self-evaluated health status, which suggests that drinking frequency and salt consumption are proper identifying variables.

**Table 3:** *T*-test for the differences in the various groups of self-reported health status in the elderly.

	**(1)**	**(2)**	**(3)**	**(4)**	**(5)**	**(6)**
	**Excellent or good**	**Fair**	**Poor**	**Diff. (1)–(3)**	**Diff. (1)–(2)**	**Diff. (2)–(3)**
*Laborhour*	330.461	317.605	204.914	125.547***	12.855	112.692***
*Age*	65.215	66.167	67.606	−2.391***	−0.952***	−1.439***
*Male*	0.420	0.364	0.364	0.055**	0.055***	0
*Edu*	2.997	2.635	2.244	0.753***	0.362***	0.391**
*Marr1*	0.033	0.024	0.036	−0.003	0.009	−0.012
*Marr2*	0.234	0.218	0.263	−0.03	0.016	−0.046**
*Marr3*	0.719	0.748	0.681	0.038	−0.029*	0.067***
*Hhinc_cpi*	9.136	9.079	8.852	0.284***	0.058	0.226***
*Hwealth*	0.469	0.387	0.396	0.073	0.082**	−0.009
*Drink_fre*	0.591	0.513	0.374	0.216***	0.077*	0.139**
*Salt*	5.192	5.304	5.436	−0.244*	−0.112	−0.132

*The differences in the various groups of self-reported health status in the elderly. The variables are introduced in Table 1.*

*, **, and ***indicate statistical significance at the 10, 5, and 1% levels, respectively.

### Modeling and Estimation Strategies

#### Simultaneous Equations of Health and Elderly's Labor Supply

##### The Equation of Labor Supply

For the labor supply equation in simultaneous equations, we set the Tobit model as follows:


(1) $$\begin{array}{l} \;\text{Laborhour}=\max\{0,\;\text{Laborhou}r^{*}\}=\\ \max\{0,\;\gamma_{0}\text{+}\text{Control}s^{\prime }\delta_{1}+a_{1}\text{Health}+\mu_{1}\} \end{array}$$

Where Laborhour^*^ is a latent variable, which can be interpreted as the elderly's actual willingness to work. We define the explained variable *Laborhour* as the observed labor supply, which is the actual working hours when Laborhour^*^ >0, while *Laborhour* is 0 when Laborhour^*^ ≤ 0, since we observe no actual working hours in this condition. In this sense, the data of the elderly's labor supply are censored to zero and the variable *Laborhour* is the Tobit-type limited dependent variable.

The variable *Health* representing an individual's health level is the key explanatory variable. We focused on the core coefficient α_1_, which indicates the effect of health on the elderly's labor supply. If α_1_ > *0*, it means that the worse the health condition of the elderly, the more working hours they provide, since the higher value of the variable *Health* indicates a worse health condition (individual with hypertension or poorer self-reported health).

The vector *Controls* includes variables such as the age and its square (*Age, Age2*), gender (*Male*), education levels (*Edu*), two dummies of marital status (*Marr1, Marr2*), household income (*Hhinc*) and household wealth (*Hwealth*) of the elderly, and 3-year dummies, which meets the assumption *Cov*(*Controls*, μ_1_) = 0. δ_1_ is the vector of coefficients of *Controls*, and γ_0_is the constant term. μ_1_is the random disturbance term, which meets the assumption $Var\left(\mu_{1}\right)=\;\sigma_{1}^{2}$.

The variable *Health* may be endogenous due to its correlation with the disturbance term μ_1_, since the health status may be related to some unobservable factors affecting the elderly's willingness to work. Therefore, we cannot use the general method to estimate the Tobit model because the results will be biased and inconsistent.

##### The Equation of Health (Hypertension)

For the health equation in simultaneous equations, we set the health as a binary variable as in model (2). We used *Hypertension* to represent *Health*_1_of model (2) in estimation.


(2) $$\begin{array}{l} \text{Healt}h_{1}=1\{\text{Healt}h_{1}^{*}>0\}=1\{\beta_{0}+\text{Control}s^{\prime }\;\delta_{21}+\\ \text{Identif}y^{\prime }\;\delta_{22}+v_{2}>0\}\equiv 1\;\left\{\beta_{0}+\text{Varb}s^{\prime }\delta_{2}+v_{2}>0\right\} \end{array}$$

Where 1{.} is the characteristic function, which means that, when $Health_{1}^{*}>0$, *Health*_1_ equals to 1 but is otherwise 0. $Health_{1}^{*}$ is the latent variable, which can be interpreted as the actual health condition, and *Health*_1_is the observed health. If $Health_{1}^{*}$ is >0, we can observe that the individual has hypertension diagnosed as 1 but is otherwise 0.

The vector of control variables *Varbs* includes the vectors *Controls* and *Identify*, which satisfies the assumption *Cov*(*Varbs, v*_2_) = 0. *Controls* represents the individual and family factors, which is the same as that in the labor equation as Equation (1). *Identify* represents the identifying variables that affect health but not an individual's labor supply. Including these variables in the health equation will ensure the parameters in simultaneous equations are identified and could be estimated. δ_21_ and δ_22_ are the vector of coefficients *Controls* and *Identify*, respectively, and β_0_is the constant term. *v*_2_is the random disturbance term, and for convenience, we set that it meets the assumption *Var*(*v*_2_) = 1.

Therefore, when health is a binary variable, combining Equations 1, 2, we can obtain the following simultaneous equations:


(3) $$\begin{array}{l} \text{Laborhour}=\text{max}\{0,\;\text{Laborhou}r^{\text{*}}\}\equiv \\ \text{max}\{0,\;\gamma_{0}\text{+}\text{Control}s^{\prime }\;\delta_{1}+a_{1}\text{Healt}h_{1}+\mu_{1}\}\\ \mathrm{Healt}h_{1}=1\{\beta_{0}+\text{Control}s^{\prime }\;\delta_{21}+\\ \text{Identif}y^{\prime }\;\delta_{22}+v_{2}>0\}\equiv 1\;\left\{\beta_{0}+\text{Varb}s^{\prime }\;\delta_{2}+v_{2}>0\right\} \end{array}$$

In Equation (3), the existence of endogeneity of *Health*_1_ is determined by the correlation between μ_1_ and ν_2_. If μ_1_ and ν_2_ are not correlated, there is no endogeneity in model (3); otherwise, the variable *Health*_1_ is endogenous. Therefore, we set μ_1_ and ν_2_ as jointly normal distributions with zero mean value and their covariance matrix is as follows:


(4) $$\begin{array}{l} \text{Var}(\begin{array}{c} \mu_{1}\\ \upsilon_{2} \end{array})=\left(\begin{array}{cc} \sigma_{1}^{2} & \rho_{1}\\ \rho_{1} & 1 \end{array}\right) \end{array}$$

We also set μ_1_ = ρ_1_*v*_2_+*e*_1_, where ρ_1_ = *cov*(μ_1_, *v*_2_). When ρ_1_ = 0, *Health*_1_ is exogenous, otherwise *Health*_1_ is endogenous in model (3). In this way, the omitted variables and potential reverse causality, which may lead to the endogeneity problem, are addressed in the model settings. We assume that *e*_1_ is independent to ν_2_, and then, it can be derived that $e_{1}\sim N\left(0,\sigma_{1}^{2}-\;\rho_{1}^{2}\right)$.

Therefore, in Equation (3), we need to estimate the coefficients γ_0_, α_1_, β_0_, ρ_1_, and σ_1_, as well as the coefficient vectors δ_1_ and δ_2._ The core coefficient α_1_is what we focus on most, which indicates the effect of hypertension on the rural elderly's working hours.

##### The Equation of Health (Self-Reported Health)

In the health equation, we set the health level of the elderly as an ordered variable as in model (5). We use *Selfhealth* to represent *Health*_2_of model (5) in estimation.


(5) $$\begin{array}{l} \begin{array}{l} \text{Healt}h_{2}^{*}=\chi_{0}+\text{Control}s^{{\;}^{\prime }}\eta_{21}+\text{Identif}y^{{\;}^{\prime }}\eta_{22}+\varepsilon_{2}\equiv \\ \chi_{0}+\text{Varb}s^{{\;}^{\prime }}\eta_{2}+\varepsilon_{2}\\ \mathrm{Healt}h_{2}=\{\begin{array}{l} 1,\;-\infty \;<\mathrm{Healt}h_{2}^{*}\leq \tau_{1}\;\\ 2,\;\tau_{1}<\mathrm{Healt}h_{2}^{*}\leq \tau_{2}\\ 3,\;\tau_{2}\;<\mathrm{Healt}h_{2}^{*}<+\infty \end{array} \end{array} \end{array}$$

Where $Health_{2}^{*}$ is the latent variable. Similar to Equation (2), $Health_{2}^{*}$ can be interpreted as the actual health condition, and *Health*_2_ is the correspondingly observed ordered variable with three values: 1 means the health condition is good, 2 means normal, and 3 means poor. ε_2_is the random disturbance term, and for convenience, we set that it meets the assumption *Var*(ε_2_) = 1. The other settings are the same as in Equation (2).

Therefore, when health is an ordered variable, combining Equations 1, 5, we can obtain the following simultaneous equations:


(6) $$\begin{array}{l} \text{Laborhour}=\max\left\{0,\;\text{Laborhou}r^{\text{*}}\right\}\equiv \\ \max\left\{0,\;\gamma_{0}\text{+}\text{Control}s^{{\;}^{\prime }}\delta_{1}+a_{1}\text{Healt}h_{2}+\mu_{1}\right\}\\ \text{Healt}h_{2}^{*}=\chi_{0}+\text{Control}s^{{\;}^{\prime }}\eta_{21}+\\ \text{Identif}y^{{\;}^{\prime }}\eta_{22}+\varepsilon_{2}\equiv \chi_{0}+\text{Varb}s^{{\;}^{\prime }}\eta_{2}+\varepsilon_{2}\\ \text{Healt}h_{2}=\{\begin{array}{l} 1,\;-\infty \;<\text{Healt}h_{2}^{*}\leq \tau_{1}\;\\ 2,\;\tau_{1}<\text{Healt}h_{2}^{*}\leq \tau_{2}\\ 3,\;\tau_{2}\;<\text{Healt}h_{2}^{*}<+\infty \end{array} \end{array}$$

In model (6), we also set μ_1_ and ε_2_ as jointly normal distribution with zero mean value, and their covariance matrix is as follows:


(7) $$\begin{array}{l} \mathrm{Var}\left(\begin{array}{c} \mu_{1}\\ \varepsilon_{2} \end{array}\right)=\left(\begin{array}{cc} \sigma_{1}^{2} & \rho_{2}\\ \rho_{2} & 1 \end{array}\right) \end{array}$$

Similarly, as model (4), we set μ_1_ = ρ_2_ε_2_+*e*_2_, where ρ_2_ = *cov*(μ_1_, ε_2_). When ρ_2_ = 0, *Health*_2_ is exogenous, otherwise, it is endogenous in model (6). We also assume that *e*_2_ is independent of ε_2_ and derive that $e_{2}\sim N\left(0,\sigma_{1}^{2}-\rho_{2}^{2}\right)$. The same as above, in Equation (6), we will estimate the coefficients γ_0_, α_1_, χ_0_, ρ_2_, and σ_1_, as well as the coefficient vectors δ_1_ and η_2._We focus mostly on α_1_, since it reveals how self-reported health affects the rural elderly's labor supply.

#### The Estimation

There are two main methods to estimate simultaneous equations as model (3) and model (6). The first is the full information maximum likelihood (FIML) estimation method. By maximizing the logarithmic likelihood function of the joint distribution *f*(*Labor, Health*|*Varbs*), we obtained the consistent and efficient estimation results of the parameters. Another method is the limited information maximum likelihood (LIML) method as a two-stage estimation. We will introduce the two methods, respectively, in the following sections.

##### Full Information Maximum Likelihood (FIML) Estimation

We used the binary variable and the ordered variable to measure health; therefore, we derived the FIML functions of these two cases, respectively.

When we set health as a binary variable, we have $e_{1}\sim N\left(0,\sigma_{1}^{2}-\rho_{1}^{2}\right)$, and


(8) $$\begin{array}{l} \text{Laborhou}r^{\text{*}}=\gamma_{0}+\text{Control}s^{{\;}^{\prime }}\delta_{1}+a_{1}\text{Healt}h_{1}+\mu_{1}\\ =\gamma_{0}+\text{Control}s^{{\;}^{\prime }}\delta_{1}+\\ a_{1}\text{Healt}h_{1}+\rho_{1}v_{2}+e_{1} \end{array}$$

Then, we arrive at:


(9) $$\begin{array}{r} \text{Laborhou}r^{*}{|}_{\text{Varbs},v_{2}}\tilde{\;}N(\gamma_{0}+\text{Control}s^{{\;}^{\prime }}\delta_{1}+a_{1}\text{Healt}h_{1}+\\ \rho_{1}v_{2},\sigma_{1}^{2}-\rho_{1}^{2}) \end{array}$$

Given *Varbs*, we can derive the joint distribution of *Laborhour* and *Health*_1_ as follows:


(10) $$\begin{array}{l} \begin{array}{l} f\left(\text{Laborhour},\text{Healt}h_{1}|\text{Varbs}\right)\\ \text{=}{\left[\frac{1}{\sigma_{1}}\phi \left(\frac{\text{Laborhour}-\gamma_{0}-\text{Control}s^{{\;}^{\prime }}\delta_{1}-\alpha_{1}\text{Healt}h_{1}}{\sigma_{1}}\right)\right]}^{1\left\{\text{Laborhour}>0\right\}}\\ \cdot {\left[\Phi \left(\frac{\sigma_{1}^{2}\left(\beta_{0}+\text{Varb}s^{{\;}^{\prime }}\delta_{2}\right)+\rho \left(\text{Laborhour}-\gamma_{0}-\text{Control}s^{{\;}^{\prime }}\delta_{1}-\alpha_{1}\text{Healt}h_{1}\right)}{\sigma_{1}\sqrt{\sigma_{1}^{2}-\rho_{1}^{2}}}\right)\right]}^{1\left\{\text{Laborhour}>0\right\}\times 1\;\left\{\;\text{Healt}h_{1}\text{=0}\right\}\;}\\ \cdot {\left[1-\Phi \left(\frac{\sigma_{1}^{2}\left(\beta_{0}+\text{Varb}s^{{\;}^{\prime }}\delta_{2}\right)+\rho \left(\text{Laborhour}-\gamma_{0}-\text{Control}s^{{\;}^{\prime }}\delta_{1}-\alpha_{1}\text{Healt}h_{1}\right)}{\sigma_{1}\sqrt{\sigma_{1}^{2}-\rho_{1}^{2}}}\right)\right]}^{1\left\{\text{Laborhour}>0\right\}\times 1\;\left\{\;\text{Healt}h_{1}\text{=1}\right\}\;}\\ \cdot {\left[1-\Phi \left(\beta_{0}+\text{Varb}s^{{\;}^{\prime }}\delta_{2}\right)-\int_{-\infty }^{-\beta_{0}-\text{Varb}s^{{\;}^{\prime }}\delta_{2}}\Phi \left(\frac{\gamma_{0}+\text{Control}s^{{\;}^{\prime }}\delta_{1}+\alpha_{1}\text{Healt}h_{1}+\rho_{1}v_{2}}{\sqrt{\sigma_{1}^{2}-\rho_{1}^{2}}}\right)\phi \left(v_{2}\right)dv_{2}\right]}^{1\left\{\text{Laborhour}=0\right\}\times 1\;\left\{\;\text{Healt}h_{1}\text{=0}\right\}\;}\\ \cdot {\left[\Phi \left(\beta_{0}+\text{Varb}s^{{\;}^{\prime }}\delta_{2}\right)-\int_{-\beta_{0}-\text{Varb}s^{{\;}^{\prime }}\delta_{2}}^{\text{+}\infty }\Phi \left(\frac{\gamma_{0}+\text{Control}s^{{\;}^{\prime }}\delta_{1}+\alpha_{1}\text{Healt}h_{1}+\rho_{1}v_{2}}{\sqrt{\sigma_{1}^{2}-\rho_{1}^{2}}}\right)\phi \left(v_{2}\right)dv_{2}\right]}^{1\left\{\text{Laborhour}=0\right\}\times 1\;\left\{\;\text{Healt}h_{1}\text{=}1\;\right\}\;} \end{array} \end{array}$$

When we set the health as an ordered variable, following the similar excise as above, we can derive the joint distribution of *Laborhour* and *Health*_2_ as follows:


(11) $$\begin{array}{l} f\left(\text{Laborhour},\text{Healt}h_{2}|\text{Varbs}\right)\\ ={\left[\phi \left(\frac{\text{Laborhour}-\gamma_{0}-\text{Control}s^{{\;}^{\prime }}\delta_{1}-\alpha_{1}\text{Healt}h_{2}}{\sigma_{1}}\right)\right]}^{1\left\{\text{Laborhour}>0\right\}}\\ \cdot {\left[1-\Phi \left(\frac{\sigma_{1}^{2}\left(\chi_{0}+\text{Varb}s^{{\;}^{\prime }}\eta_{2}-\tau_{1}\right)+\rho \left(\text{Laborhour}-\gamma_{0}-\text{Control}s^{{\;}^{\prime }}\delta_{1}-\alpha_{1}\text{Healt}h_{2}\right)}{\sigma_{1}\sqrt{\sigma_{1}^{2}-\rho_{{\;}^{2}}^{2}}}\right)\right]}^{1\left\{\text{Laborhour}>0\right\}\times 1\left\{\text{Healt}h_{2}=1\right\}}\\ \cdot {\left[\begin{array}{c} \Phi \left(\frac{\sigma_{1}^{2}\left(\tau_{2}-\chi_{0}-\text{Varb}s^{{\;}^{\prime }}\eta_{2}\right)+\rho \left(\text{Laborhour}-\gamma_{0}-\text{Control}s^{{\;}^{\prime }}\delta_{1}-\alpha_{1}\text{Healt}h_{2}\right)}{\sigma_{1}\sqrt{\sigma_{1}^{2}-\rho_{{\;}^{2}}^{2}}}\right)\\ -\Phi \left(\frac{\sigma_{1}^{2}\left(\tau_{1}-\chi_{0}-\text{Varb}s^{{\;}^{\prime }}\eta_{2}\right)+\rho \left(\text{Laborhour}-\gamma_{0}-\text{Control}s^{{\;}^{\prime }}\delta_{1}-\alpha_{1}\text{Healt}h_{2}\right)}{\sigma_{1}\sqrt{\sigma_{1}^{2}-\rho_{{\;}^{2}}^{2}}}\right) \end{array}\right]}^{1\left\{\text{Laborhour}>0\right\}\times 1\left\{\text{Healt}h_{2}=2\right\}}\\ \cdot {\left[1-\Phi \left(\frac{\sigma_{1}^{2}\left(\tau_{2}-\chi_{0}-\text{Varb}s^{{\;}^{\prime }}\eta_{2}\right)+\rho \left(\text{Laborhour}-\gamma_{0}-\text{Control}s^{{\;}^{\prime }}\delta_{1}-\alpha_{1}\text{Healt}h_{2}\right)}{\sigma_{1}\sqrt{\sigma_{1}^{2}-\rho_{{\;}^{2}}^{2}}}\right)\right]}^{1\left\{\text{Laborhour}>0\right\}\times 1\left\{\text{Healt}h_{2}=3\right\}}\\ \cdot {\left[\Phi \left(\tau_{1}-\chi_{0}-\text{Varb}s^{{\;}^{\prime }}\eta_{2}\right)-\int_{-\infty }^{\tau_{1}-\chi_{0}-\text{Varb}s^{{\;}^{\prime }}\eta_{2}}\Phi \left(\frac{\gamma_{0}+\text{Control}s^{{\;}^{\prime }}\delta_{1}+\alpha_{1}\text{Healt}h_{2}+\rho_{2}\varepsilon_{2}}{\sqrt{\sigma_{1}^{2}-\rho_{{\;}^{2}}^{2}}}\right)\phi \left(\varepsilon_{2}\right)d\varepsilon_{2}\right]}^{1\left\{\text{Laborhour}=0\right\}\times 1\left\{\text{Healt}h_{2}=1\right\}}\\ \cdot {\left[\Phi \left(\tau_{2}-\chi_{0}-\text{Varb}s^{{\;}^{\prime }}\eta_{2}\right)-\Phi \left(\tau_{1}-\chi_{0}-\text{Varb}s^{{\;}^{\prime }}\eta_{2}\right)-\int_{\tau_{1}-\chi_{0}-\text{Varb}s^{{\;}^{\prime }}\eta_{2}}^{\tau_{2}-\chi_{0}-\text{Varb}s^{{\;}^{\prime }}\eta_{2}}\Phi \left(\frac{\gamma_{0}+\text{Control}s^{{\;}^{\prime }}\delta_{1}+\alpha_{1}\text{Healt}h_{2}+\rho_{2}\varepsilon_{2}}{\sqrt{\sigma_{1}^{2}-\rho_{{\;}^{2}}^{2}}}\right)\phi \left(\varepsilon_{2}\right)d\varepsilon_{2}\right]}^{1\left\{\text{Laborhour}=0\right\}\times 1\left\{\text{Healt}h_{2}=2\right\}}\\ \cdot {\left[1-\Phi \left(\tau_{2}-\chi_{0}-\text{Varb}s^{{\;}^{\prime }}\eta_{2}\right)-\int_{\tau_{2}-\chi_{0}-\text{Varb}s^{{\;}^{\prime }}\eta_{2}}^{+\infty }\Phi \left(\frac{\gamma_{0}+\text{Control}s^{{\;}^{\prime }}\delta_{1}+\alpha_{1}\text{Healt}h_{2}+\rho_{2}\varepsilon_{2}}{\sqrt{\sigma_{1}^{2}-\rho_{{\;}^{2}}^{2}}}\right)\phi \left(\varepsilon_{2}\right)d\varepsilon_{2}\right]}^{1\left\{\text{Laborhour}=0\right\}\times 1\left\{\text{Healt}h_{2}=3\right\}} \end{array}$$

Appendixes A1 and A2 show the detailed probability definitions and derivation process of models (10) and (11), respectively. We wrote a GAUSS program to estimate the parameters (coefficients γ_0_, α_1_, β_0_, ρ_1_, σ_1_, and the coefficients vectors δ_1_, δ_2_) in model (10) and in model (11) (coefficients γ_0_, α_1_, χ_0_, ρ_2_, σ_1_, and the coefficients vectors δ_1_, η_2_), since the standard econometric packages do not include this type of model.

##### Two-Stage Limited Information Maximum Likelihood (Two-Stage LIML) Estimation

Besides the FMIL method, we also used the two-stage LIML method as a robustness check. Using the two-stage LIML method, we can avoid the difficulties of the convergence of the estimation and the derivation of the joint distribution, while the estimation results are still consistent.

For example, when the health is a binary variable as Equation (3), compared with the FIML method, we no longer estimated the joint distribution of *Laborhour* and *Health*_1_ directly but estimated the distributions of *f*_2_(*Health*_1_|*Varbs*, β_0_, δ_2_, σ_1_) and *f*_1_(*Laborhour*|*Varbs*, β_0_, δ_2_, σ_1_, γ_0_, α_1_, δ_1_, ρ_1_) by two steps. In the first step, we used the maximum likelihood method to get the estimated parameters in the health equation ($\hat{\beta_{0}},\;\hat{\delta_{2}},\;\hat{\sigma_{1}}$), The conditional likelihood function is:


(12) $$\begin{array}{l} ll_{2}=\overset{N}{\underset{i=1}{\sum }}\ln\;f_{2}\left(\text{Healt}h_{1}|\text{Varbs},\beta_{0},\delta_{2},\sigma_{1}\right) \end{array}$$

In the second step, we took $\hat{\beta_{0}}$ and $\hat{\delta_{2}}$ to construct $He\hat{a}lth_{1})$ and used the maximum likelihood method to estimate parameters in the labor equation, and the conditional likelihood function is:


(13) $$\begin{array}{l} ll_{1}=\overset{N}{\underset{i=1}{\sum }}\ln\;f_{1}\left(\text{Laborhour}|\text{Varbs},\beta_{0},\delta_{2},\sigma_{1},\gamma_{0},\alpha_{1},\delta_{1},\rho_{1}\right) \end{array}$$

It can be proved that all the estimated parameters are consistent estimators. However, the problem with the two-stage LIML method is that, in the second step, the predicted value of health ($Hea\hat{l}th_{1})$ is taken into the labor equation, and then the prediction error will affect the disturbance term of the labor equation and cause bias; thus, we need to correct the prediction error. Specifically, we estimated the covariance matrix (*V*) following Murphy and Topel (31), which presents a correction method of the asymptotic covariance matrix in the two-stage estimation. We set the unknown parameter vector $\theta_{1}={\left(\gamma_{0},\alpha_{1},\delta_{1},\rho_{1}\right)}^{{\;}^{\prime }}$ and $\theta_{2}={\left(\beta_{0},\delta_{2},\sigma_{1}\right)}^{{\;}^{\prime }}$, and then the asymptotic covariance matrix (*V*) of θ_1_ is:


(14) $$\begin{array}{l} V=V_{1}+V_{1}\left(CV_{2}C^{\prime }-RV_{2}C^{\prime }-CV_{2}R^{\prime }\right)V_{1} \end{array}$$

where $V_{2}=Asy.Var\left(\hat{\theta_{2}}\right)$, which is obtained by the estimation of *ll*_2_, $V_{1}=Asy.Var\left(\hat{\theta_{1}}\right)$ obtained by the estimation of *ll*_1_, $C=E\left\{\left(\frac{\partial u1}{\partial \theta_{1}}\right)\left(\frac{\partial u1}{\partial \theta_{2}^{{\;}^{\prime }}}\right)\right\}$ and $R=E\left\{\left(\frac{\partial u1}{\partial \theta_{1}}\right)\left(\frac{\partial u2}{\partial \theta_{2}^{{\;}^{\prime }}}\right)\right\}$. The detailed proofs are in Murphy and Topel (31).

Besides, according to Cameron and Trivedi (32), we used another bootstrap method to correct the standard error in the two-stage estimation. The bootstrap method is based on the empirical distribution; so it is easier to obtain a consistent estimation of the standard error without the correction step in non-linear models (32). When estimating Equation (6) where the health is an ordered variable, we used the same LIML estimation method.

## Results

We first tested the endogeneity of the variables *Health*_1_ and *Health*_2_ to decide whether we need to address the endogeneity problem when estimating the simultaneous equations. Then, we analyzed the results to see the impact of health on the elderly's labor supply in rural areas.

### The Endogeneity Test

Whether the variables *Health*_1_ and *Health*_2_ in models (3) and (6) are endogenous can be determined by testing the significance of ρ_1_ and ρ_2_, which represents the correlations of the disturbance terms in the labor equation and the health equation. For example, in model (3), if the null hypothesis ρ_1_ = *cov(*μ_1_ , ν_2_*)* = 0, it means that there is no endogeneity; otherwise, *Health*_1_ is endogenous. Following Wooldridge (33), the test of model (3) includes two steps: First, we estimated the health equation and got the predicted residual $\hat{\nu_{2}}$. Second, we estimated the labor equation by taking $\hat{\nu_{2}}$ into it and then using *t*-test to determine whether ρ_1_ is significantly different from zero.

The test results show that when health is the binary variable as hypertension diagnosed or not, the *t*-value is −2.16 and the *p*-value is 0.031. For the ordered variable as self-reported health, the *t*-value is −3.55 and the *p*-value is 0. This implies that the variables *Health*_1_ and *Health*_2_ in models (3) and (6) are endogenous. Therefore, in the following section, we will use the FIML and LIML estimation methods to examine the effect of health on the elderly's labor supply.

### The Effect of Health on the Elderly's Labor Supply

Table 4 shows the estimation results of the simultaneous equations using the binary variable *Health*_1_. We found that the coefficients of *Health*_1_ are all negative but not significant in the labor equations [columns (2), (4), (5) and (6)], which means that the failing health does not significantly decrease the elderly's labor supply in rural China. Our finding indicates the phenomenon of “ceaseless toil” for the elderly in rural China, i.e., the elderly almost work their whole life even if they are not physically capable.

**Table 4:** The effect of health on the elderly's labor supply (using the objective indicator of health: Hypertension diagnosed or not).

	**(1)**	**(2)**	**(3)**	**(4)**	**(5)**	**(6)**
	**FIML**	**FIML**	**LIML**	**LIML**	**LIML**	**LIML**
	**Health equation**	**Labor equation**	**Health equation**	**Labor equation (Original std.)**	**Labor equation (MT std.)**	**Labor equation (Bootstrap std.)**
*Hypertension*		−0.198		−0.084	−0.084	−0.084
		(1.672)		(1.569)	(1.567)	(2.032)
*Age*	0.036	0.824	0.109**	0.894***	0.894***	0.894***
	(0.112)	(0.846)	(0.049)	(0.185)	(0.185)	(0.216)
*Age2*	0.000	−0.000*	−0.001	−0.008***	−0.008***	−0.008***
	(0.001)	(0.000)	(0.000)	(0.001)	(0.001)	(0.002)
*Male*	0.054	0.850***	0.098*	0.893***	0.893***	0.893***
	(0.122)	(0.160)	(0.056)	(0.180)	(0.179)	(0.194)
*Edu*	−0.005	−0.061***	−0.011	−0.119***	−0.119***	−0.119***
	(0.017)	(0.022)	(0.008)	(0.026)	(0.026)	(0.029)
*Marr1*	0.011	−0.231	0.035	−0.224	−0.224	−0.224
	(0.303)	(0.378)	(0.133)	(0.427)	(0.426)	(0.437)
*Marr2*	0.014	−0.528***	0.028	−0.837***	−0.837***	−0.837***
	(0.129)	(0.168)	(0.057)	(0.186)	(0.186)	(0.250)
*Hhinc*	−0.017	0.195***	−0.013	0.482***	0.482***	0.482***
	(0.048)	(0.060)	(0.021)	(0.069)	(0.069)	(0.076)
*Hwealth*	−0.033	−0.044	−0.005	−0.344***	−0.344***	0.344***
	(0.052)	(0.072)	(0.023)	(0.076)	(0.076)	(0.081)
*Drink_fre*	0.004		0.000			
	(0.045)		(0.021)			
*Salt*	−0.068		−0.099***			
	(0.066)		(0.028)			
*Salt2*	0.006		0.008***			
	(0.004)		(0.002)			
*Constant*	−1.523	−20.577***	−4.579***	−21.661***	−21.661***	−21.661***
	(3.823)	(4.964)	(1.684)	(6.269)	(6.266)	(7.161)
*Year effect*	Yes	Yes	Yes	Yes	Yes	Yes
*N*	3,535	3,535	3,535	3,535	3,535	3,535

*The estimates of the effect of health on the elderly's labor supply using the objective indicator hypertension. Columns (1) and (2) present the results of the FIML estimation and columns (3) to (6) are the results of the two-stage LIML estimation. The standard errors in column (4) are the original ones in estimation, in column (5) are corrected following the method of Murphy and Topel (31), and in column (6) are corrected by the bootstrap method. The explained variable is the annual working hours of the elderly in rural China (lnLaborhour). The explanatory variables are introduced in Table 1. Standard errors are reported in parentheses below the coefficients.*

*, **, and ***indicate statistical significance at the 10, 5, and 1% levels, respectively.

For the robustness of the results, we provided both the FIML and two-stage LIML estimation results with three kinds of corrected standard errors. For the size of the standard errors with three methods [column (4)–(6)], the original standard error without correction and the standard error corrected following the method of Murphy and Topel (31) were similar, while the standard error corrected by the bootstrap method seems significantly greater than the other two ones. However, the differences in standard errors do not affect the robustness of our results.

For the control variables, first, the coefficients of *Age2* were significantly negative, which means that the relationship between age and labor supply is non-linear as the inverted U shape. Second, the coefficients of *Male* are significantly positive in labor equations, which reveal that elderly men work more hours than women. This is consistent with the studies of Wang (34), Ling and Chi (35). For this reason, the elderly men in rural areas spend more working hours in family agricultural production. Third, the significantly negative coefficients of the variable *Edu* in labor equations indicate that the elderly with higher education levels work less. They may have better jobs and positions and enjoy higher welfare, so they tend to spend less time working when getting older. Forth, for the coefficients of household income (*Hhinc*) and wealth (*Hwealth*), we found that the income significantly increases the labor supply while the wealth lowers it. It means that the elderly work more to increase family income, but if the family is rich in assets, they may work less.

Table 5 shows the estimation results of the effect of self-reported health on the elderly's labor supply. In column (2), it shows that health does not significantly affect the working hours of the elderly in rural China when using the FIML estimation method. We found that there is a positive correlation between self-reported health and working hours, which means the poorer the self-evaluated health, the longer the working hours. Although the positive correlation is not statistically significant, it also indicates the phenomenon of “ceaseless toil” of the elderly from a subjective measurement of health.

**Table 5:** The effect of health on the elderly's labor supply (using the subjective indicator of health: Self-reported health).

	**(1)**	**(2)**	**(3)**	**(4)**	**(5)**	**(6)**
	**FIML**	**FIML**	**LIML**	**LIML**	**LIML**	**LIML**
	**Health equation**	**Labor equation**	**Health equation**	**Labor equation (Original std.)**	**Labor equation (MT std.)**	**Labor equation (Bootstrap std.)**
*Selfhealth*		5.345		7.465**	7.465	7.465
		(5.513)		(2.933)	(7.478)	(5.781)
*Age*	0.040***	0.063	0.097**	1.165***	1.165	1.165***
	(0.009)	(0.120)	(0.042)	(0.219)	(0.767)	(0.341)
*Age2*	−0.000	−0.001**	−0.001**	−0.010***	−0.010*	−0.010***
	(0.001)	(0.000)	(0.000)	(0.002)	(0.005)	(0.002)
*Male*	−0.066***	0.810***	−0.121**	0.466*	0.466	0.466
	(0.023)	(0.172)	(0.049)	(0.247)	(1.008)	(0.427)
*Edu*	−0.008	−0.054**	−0.022***	−0.180***	−0.180**	−0.180
	(0.030)	(0.022)	(0.007)	(0.035)	(0.080)	(0.068)
*Mar1*	−0.018	−0.377	−0.016	−0.260	−0.260	−0.26
	(0.501)	(0.347)	(0.117)	(0.426)	(0.588)	(0.678)
*Mar2*	−0.050	−0.306*	−0.106**	−1.144***	−1.144	−1.144***
	(0.240)	(0.165)	(0.050)	(0.238)	(0.731)	(0.343)
*Hhinc*	−0.047	0.217**	−0.051***	0.332***	0.332	0.332***
	(0.089)	(0.087)	(0.018)	(0.098)	(0.289)	(0.142)
*Hwealth*	−0.007	−0.101***	−0.011	−0.379***	−0.379***	−0.379***
	(0.094)	(0.004)	(0.021)	(0.081)	(0.120)	(0.097)
*Drink_fre*	−0.018**		−0.040**			
	(0.007)		(0.018)			
*Salt*	−0.016		−0.034			
	(0.096)		(0.023)			
*Salt2*	0.001**		0.003**			
	(0.000)		(0.001)			
*Cut1*	2.001***		3.114**			
	(0.771)		(1.448)			
*Cut2*	4.171***		4.521***			
	(1.101)		(1.449)			
*Constant*		15.109		−34.265***	−34.265	−34.265
		(15.018)		(8.130)	(27.961)	(13.604)
*Year effect*	Yes	Yes	Yes	Yes	Yes	Yes
*N*	3,535	3,535	3,535	3,535	3,535	3,535

*The estimates of the effect of health on the elderly's labor supply using the subjective indicator self-reported health. Columns (1) and (2) present the results of the FIML estimation and columns (3) to (6) are the results of the two-stage LIML estimation. The standard errors in column (4) are the original ones in estimation, in column (5) are corrected following the method of Murphy and Topel (31), and in column (6) are corrected by the bootstrap method. The explained variable is the annual working hours of the elderly in rural China (lnLaborhour). The explanatory variables are introduced in Table 1. Standard errors are reported in parentheses below the coefficients.*

*, **, and ***indicate statistical significance at the 10, 5, and 1% levels, respectively.

However, the results of the two-stage LIML estimation are shown inconsistently in columns (4) to (6). It is worth mentioning that if we use the original standard error in column (4) to make a statistical inference, the effect of health on labor supply is significant at a 5% level. However, we cannot give enough credit to this empirical result because the standard error has not been corrected. As we introduced in Section “The Estimation”, the problem of the two-stage LIML method is that the prediction error will affect the disturbance term of the labor equation and cause estimation bias. If we do not correct the prediction error, the hypothesis tests based on the estimated covariance matrix of the second-step estimator are biased (31).

Therefore, in columns (5) and (6), we use the Murphy and Topel (31) standard error and the bootstrapped standard error, respectively, and the significance disappears. This also indicates that the correction and robustness check are necessary in two-stage estimation.

## Discussion

### The Effect of Health on the Elderly's Labor Supply

The effect of health on the elderly's labor supply is controversial in the literature. While some studies found that health has a significant positive impact on labor supply (6, 18–20, 36), some other studies found that there is no significant effect (3, 21–24). Benjamin et al. (3) and Tan and Zhou (22) hold that there is the phenomenon of “ceaseless toil” existing among the elderly in rural China. French (21) found that the declining health explains only a small fraction of retirement, and the number of unhealthy adults above age 55 is far smaller than the number of those who drop their employment. Coile et al. (23) also found that the declines in health are much too small to explain the declines in the employment of older people.

The reason for the controversy is two-fold. One is that the literature varies in the ways in dealing with the endogeneity problem of health variables. In Section “The Endogeneity Problem of the Health,” we will discuss it in detail. The second is about the measurements of labor supply. Most studies focus on labor participation rather than labor hours (18–20, 37). Since people always choose to work either full time or not at all, this may lead to a significant effect of health on labor participation but not on labor hours. In this study, we addressed this issue in two ways. First, the sample we used is the rural older adults who are mainly self-employed and therefore they have a more flexible working hours. Second, the equation of labor supply we used is a Tobit model, which takes into account both the continuous working hours and zero working hours to avoid the estimation bias.

Therefore, by using the simultaneous-equation Tobit model with both the subjective and objective health indicators, this study adds to the literature on the causal effect of health on the elderly's labor supply and in particular to the few studies that examine the effect in developing countries. We found that failing health does not significantly decrease the elderly's labor supply in rural areas. The results further support the conclusion of Benjamin et al. (3) that there is a phenomenon of “ceaseless toil” among the elderly in rural areas in China. That is to say that the rural elderly almost work their whole life even if they are not physically capable.

### The Endogeneity Problem of the Health

Alleviating the endogeneity problem of health variables is essential for examining the effect of health on the elderly's labor supply. Some studies used self-reported health status to measure health and found that it is strongly associated with early retirement (38–41). However, as a subjective health indicator, the self-reported health status is susceptible to the influence of an individual's heterogeneous characteristics, which may cause omitted variables and measurement errors (25).

Other studies used objective health indicators as the instrumental variables for health, such as hospital stay, BMI index, parental health indicators, and health behaviors, such as current or past smoking experience, physical exercise frequency, and alcohol abuse (9, 10). However, the information advantage of subjective health indicators may be lost and lead to a weak-instruments problem. In addition, some studies directly investigate the impact of objective health indicators on labor supply. Kalwij and Vermeulen (11) used the physical function limitations to measure health, but the study has some drawbacks as the physical function limitations tend to occur in the senior elderly. The young elderly, however, as the main participants of the elderly's labor supply, are less likely to have physical function limitations.

Some scholars used both objective and subjective health indicators in their studies. Mete and Schultz (6) analyzed the effect of health on the elderly's labor participation in Taiwan using three types of health indicators as self-reported health, ADLs, and specific diseases. Besides, some researchers used principal component analysis to construct composite health indicators (42, 43). However, such composite indicators may lack economic meaning and become inaccurate when single indicators are not highly correlated.

In this study, we used both objective and subjective health indicators, i.e., hypertension and self-reported health status. In the simultaneous-equation Tobit model, we emphasized hypertension as a binary variable by setting the equation of health as a Probit model and self-reported health as an ordered variable by setting an ordered Probit model. In this way, we investigated the effect of health on the elderly's labor supply from different perspectives and enriched the measures of health in the literature.

### The Estimation Strategies

To address the endogeneity problem of the variable health in estimation, some studies used the method of instrumental variables. However, it is very difficult to find a proper instrumental variable that can affect health but not affect labor supply through channels except health. Scholars mainly used objective health indicators as the instrumental variables for subjective health indicators. For example, Campolieti (9) studied the impact of physical disability on labor force participation among the Canadian elderly using specific disease indicators and BMI as the instrumental variables for physical disability. Sheran (44) used regional prices of food and health service as instrumental variables for subjective health indicators. Latif (45) used health information as diabetes diagnosed or not of the individual's father, mother, and siblings as the instrumental variables for the individual's own diabetes.

Some studies used simultaneous equations to address the endogeneity problem (10, 46–48). Different from the single equation model usually estimated by two-stage least squares, in simultaneous equations, the correlation of the residuals between the labor equation and the health equation is sufficiently considered. Therefore, the omitted variables and reverse causality that may lead to the endogeneity problem are addressed in simultaneous equations.

In general, the simultaneous equations are usually estimated by the two-stage LIML or the FIML method. Essentially, the two-stage LIML method is the instrumental variable method, and each equation is estimated separately using exogenous instruments. We can obtain a consistent but not efficient estimator using this method because the correlation of residuals in simultaneous equations is not fully taken into consideration. However, the FIML method fully considers the correlation of residuals, i.e., the unobserved factors affect both labor and health simultaneously. Thus, the estimation results are not only consistent but also efficient.

In addition, using the FIML method, the correlation coefficient of residuals in the labor equation and the health equation can be directly estimated, and we can test the significance of the correlation coefficient directly to verify the existence of endogeneity. If we used the two-stage LIML estimation, the endogeneity can only be partially tested based on the second step in the labor Equation (10). Using the FIML method, Cai and Kalb (10) estimated the simultaneous equations of the binary variable (labor participation) and the ordered variable (health) and analyzed the impact of self-reported health on labor participation of Australian adults. Zhang et al. (46) estimated the simultaneous equations of five simultaneous binary variables using the FIML method and investigated the effect of diabetes, cardiovascular disease, mental illness, and other chronic diseases on labor force participation in Australia.

The difference between our simultaneous equations model and the previous ones is that, in the labor equation, our explained variable is a Tobit-type limited dependent variable rather than a binary variable. In the health equation, we used two types of variables to measure the health level, the binary variable (hypertension diagnosed or not) and the ordered variable (self-reported health status). Based on these variables, we derived the logarithmic likelihood function of joint distribution and then used the FIML method to estimate the results. As we could not find the literature that use FIML method to estimate simultaneous equations of this type, our study is a beneficial supplement to current literature.

## Conclusion

In this study, we used the four-period CHNS data in the years 1997, 2001, 2004, and 2006 to estimate the impact of health on the working hours of the elderly in rural China. The results showed that neither the objective indicator (hypertension diagnosed or not) nor the subjective indicator (self-reported health status) has a significant impact on the labor supply of the rural elderly. This means that the rural residents have to keep working even if they are suffering from chronic diseases or failing health. We focused on the endogeneity of health variables in the Tobit model of working hours and used the FIML and two-stage LIML estimation methods to conduct simultaneous estimations of labor supply (Tobit model) and health (Probit model and Ordered-Probit model) equations. Our estimation strategy can be used for further empirical research of relevant models.

The split between the urban and rural social security systems in China has lasted for a long time. The conflict between labor supply and the health welfare of the elderly living in rural China raises a lot of compelling questions. For instance, how to better design the social security system in rural China? Due to the reduced fertility rates and increasing medical costs, many countries have great fiscal pressures and seek policy reforms for the later retirement of older people (24). In rural China, these policy reforms may not be applicable. Our study is meaningful to policymakers for the medical and retirement policies in China.

## Data Availability Statement

Publicly available datasets were analyzed in this study. This data can be found here: https://www.cpc.unc.edu/projects/china.

## Author Contributions

All authors listed have made a substantial and intellectual contribution to the work, and approved it for publication.

## Funding

We acknowledge the financial support from the Humanities and Social Science Fund of Ministry of Education of China (Grant No: 18YJC790146), National Natural Science Foundation of China (Grant Nos: 71903042 and 72003050), Natural Science Foundation of Guangdong Province (Grant Nos: 2019A1515110196 and 2020A1515010434), and Guangzhou Philosophy and Social Science Planning Project (2021-GJ-02).

## Conflict of Interest

The authors declare that the research was conducted in the absence of any commercial or financial relationships that could be construed as a potential conflict of interest.

## Publisher's Note

All claims expressed in this article are solely those of the authors and do not necessarily represent those of their affiliated organizations, or those of the publisher, the editors and the reviewers. Any product that may be evaluated in this article, or claim that may be made by its manufacturer, is not guaranteed or endorsed by the publisher.

## Footnotes

^1^We used the above four years because the questions of self-reported health status were surveyed only in these four years.

^2^China has implemented a compulsory regulation of retirement age in urban areas since the 1950s, with 60 for male and 55 for female professionals or cadres (including teachers, medical personnel, other professionals, and administrators), and 50 for the rest of the female workers (2). Correspondingly, in this study, we used a narrower sample by defining the age 55 or above as the elderly for women in rural areas.
