# Supplementary material for: The Effect of Health on the Elderly's Labor Supply in Rural China: Simultaneous Equation Models With Binary, Ordered, and Censored Variables
Source: Front Public Health. 2022 Jul 13;10:890374. doi: 10.3389/fpubh.2022.890374 (PMC9326090; doi:10.3389/fpubh.2022.890374)
Supplement: Supplementary file 1 [file Data_Sheet_1.PDF]

Appendix

A1. FIML for the binary variable of health (Hypertension)

When the health is a binary variable, we have the following simultaneous equations:

$$\begin{aligned} Laborhour &= \max\{0, Laborhour^*\} \equiv \max\{0, \gamma_0 + Controls'\delta_1 + a_1 Health_1 + \mu_1\} \\ Health_1 &= 1\{\beta_0 + Controls'\delta_{21} + Identify'\delta_{22} + v_2 > 0\} \equiv 1\left\{\beta_0 + Varbs'\delta_2 + v_2 > 0\right\} \end{aligned} \quad (4)$$

where $Cov(Varbs,\ v_2) = 0$, $Var\begin{pmatrix}\mu_1 \\ v_2\end{pmatrix} = \begin{pmatrix}\sigma_1^2 & \rho_1 \\ \rho_1 & 1\end{pmatrix}$, and $\mu_1 = \rho_1 v_2 + e_1$, $\rho_1 = cov(\mu_1, v_2)$. μ_1 and v_2 are jointly normal distribution with zero mean value.

Since we have $e_1 \sim N(0, \sigma_1^2 - \rho_1^2)$, and

$$\begin{aligned} Laborhour^* &= \gamma_0 + Controls'\delta_1 + a_1 Health_1 + \mu_1 \\ &= \gamma_0 + Controls'\delta_1 + a_1 Health_1 + \rho_1 v_2 + e_1 \end{aligned} \quad (A.1)$$

Then we arrive at:

$$Laborhour^* |_{Varbs, v_2} \sim N(\gamma_0 + Controls'\delta_1 + a_1 Health_1 + \rho_1 v_2, \sigma_1^2 - \rho_1^2) \quad (A.2)$$

Given *Varbs*, the joint distribution of $Laborhour^*$ and $Health_1$ could be derived as:

$$f(Laborhour^*, Health_1 | Varbs) = f(Laborhour^* | Health_1, Varbs) \times f(Health_1 | Varbs) \quad (A.3)$$

If *Laborhour* = 0, we have:

$$\begin{aligned} & P(Laborhour = 0, Health_1 = 1 | Varbs) \\ &= E\left[E\left(1\{Laborhour^* \le 0\} | v_2, Varbs\right) | Health_1 = 1, Varbs\right] P(Health_1 = 1 | Varbs) \\ &= E\left[P\left(e_1 \le -\gamma_0 - Controls'\delta_1 - a_1 Health_1 - \rho_1 v_2 | v_2, Varbs\right) | Health_1 = 1, Varbs\right] P(Health_1 = 1 | Varbs) \\ &= E\left[\Phi\left(-\frac{\gamma_0 + Controls'\delta_1 + a_1 Health_1 + \rho_1 v_2}{\sqrt{\sigma_1^2 - \rho_1^2}}\right) | v_2 > -\beta_0 - Varbs'\delta_2, Varbs\right] P(Health_1 = 1 | Varbs) \\ &= \int_{-\beta_0 - Varbs'\delta_2}^{+\infty} \Phi\left(-\frac{\gamma_0 + Controls'\delta_1 + a_1 Health_1 + \rho_1 v_2}{\sqrt{\sigma_1^2 - \rho_1^2}}\right) \phi(v_2) dv_2 \\ &= \Phi(\beta_0 + Varbs'\delta_2) - \int_{-\beta_0 - Varbs'\delta_2}^{+\infty} \Phi\left(\frac{\gamma_0 + Controls'\delta_1 + a_1 Health_1 + \rho_1 v_2}{\sqrt{\sigma_1^2 - \rho_1^2}}\right) \phi(v_2) dv_2 \end{aligned}$$

(A.4)

$$P(Laborhour=0, Health_1=0\,|\,Varbs)$$
$$=E\Big[E\big(1\{Laborhour^*\le 0\}\,|\,v_2,Varbs\big)\,|\,Health_1=0,Varbs\Big]P(Health_1=0\,|\,Varbs)$$
$$=E\Big[P\big(e_1\le -\gamma_0-Controls'\delta_1-a_1Health_1-\rho_1v_2\,|\,v_2,Varbs\big)\,|\,Health_1=0,Varbs\Big]P(Health_1=0\,|\,Varbs)$$
$$=E\left[\Phi\left(-\frac{\gamma_0+Controls'\delta_1+a_1Health_1+\rho_1v_2}{\sqrt{\sigma_1^2-\rho_1^2}}\right)|\,v_2\le -\beta_0-Varbs'\delta_2,Varbs\right]P(Health_1=0\,|\,Varbs)$$
$$=\int_{-\infty}^{-\beta_0-Varbs'\delta_2}\Phi\left(-\frac{\gamma_0+Controls'\delta_1+a_1Health_1+\rho_1v_2}{\sqrt{\sigma_1^2-\rho_1^2}}\right)\phi(v_2)dv_2$$
$$=1-\Phi(\beta_0+Varbs'\delta_2)-\int_{-\infty}^{-\beta_0-Varbs'\delta_2}\Phi\left(\frac{\gamma_0+Controls'\delta_1+a_1Health_1+\rho_1v_2}{\sqrt{\sigma_1^2-\rho_1^2}}\right)\phi(v_2)dv_2$$

(A.5)

If $Laborhour = Laborhour^*$, for any $Laborhour>0$, we have:

$$P(Laborhour, Health_1=1|Varbs)$$
$$=E\Big[E\big(1\{Laborhour^*<Laborhour\}\,|\,v_2,Varbs\big)\,|\,Health_1=1|Varbs\Big]P(Health_1=1|Varbs)$$
$$=E\Big[P\big(e_1<Laborhour-\gamma_0-Controls'\delta_1-a_1Health_1-\rho_1v_2\,|\,v_2,Varbs\big)\,|\,Health_1=1,Varbs\Big]P(Health_1=1|Varbs)$$
$$=E\left[\Phi\left(\frac{Laborhour-(\gamma_0+Controls'\delta_1+a_1Health_1+\rho_1v_2)}{\sqrt{\sigma_1^2-\rho_1^2}}\right)|\,v_2>-\beta_0-Varbs'\delta_2,Varbs\right]P(Health_1=1|Varbs)$$
$$=\int_{-\beta_0-Varbs'\delta_2}^{+\infty}\Phi\left(\frac{Laborhour-(\gamma_0+Controls'\delta_1+a_1Health_1+\rho_1v_2)}{\sqrt{\sigma_1^2-\rho_1^2}}\right)\phi(v_2)dv_2$$

(A.6)

$$P(Laborhour, Health_1=0\,|\,Varbs)$$
$$=E\Big[E\big(1\{Laborhour^*<Laborhour\}\,|\,v_2,Varbs\big)\,|\,Health_1=0,Varbs\Big]P(Health_1=0\,|\,Varbs)$$
$$=E\Big[P\big(e_1<Laborhour-\gamma_0-Controls'\delta_1-a_1Health_1-\rho_1v_2\,|\,v_2,Varbs\big)\,|\,Health_1=0,Varbs\Big]P(Health_1=0\,|\,Varbs)$$
$$=E\left[\Phi\left(\frac{Laborhour-(\gamma_0+Controls'\delta_1+a_1Health_1+\rho_1v_2)}{\sqrt{\sigma_1^2-\rho_1^2}}\right)|\,v_2\le -\beta_0-Varbs'\delta_2,Varbs\right]P(Health_1=0\,|\,Varbs)$$
$$=\int_{-\infty}^{-\beta_0-Varbs'\delta_2}\Phi\left(\frac{Laborhour-(\gamma_0+Controls'\delta_1+a_1Health_1+\rho_1v_2)}{\sqrt{\sigma_1^2-\rho_1^2}}\right)\phi(v_2)dv_2$$

(A.7)

Therefore, when $Laborhour>0$, we arrive at:

$$f(Laborhour, Health_1 = 1 | Varbs)$$

$$= \frac{1}{\sqrt{\sigma_1^2 - \rho_1^2}} \int_{-\beta_0 - Varbs'\delta_2}^{+\infty} \phi\left(\frac{Laborhour - (\gamma_0 + Controls'\delta_1 + a_1 Health_1 + \rho_1 v_2)}{\sqrt{\sigma_1^2 - \rho_1^2}} \right) \phi(v_2) dv_2$$

$$= \frac{1}{\sqrt{\sigma_1^2 - \rho_1^2}} \int_{-\beta_0 - Varbs'\delta_2}^{+\infty} \frac{1}{2\pi} \cdot e^{-\frac{\left(\frac{Laborhour - (\gamma_0 + Controls'\delta_1 + a_1 Health_1 + \rho_1 v_2)}{\sqrt{\sigma_1^2 - \rho_1^2}}\right)^2}{2}} * e^{-\frac{v_2^2}{2}} dv_2$$

$$= \frac{1}{\sqrt{\sigma_1^2 - \rho_1^2}} \int_{-\beta_0 - Varbs'\delta_2}^{+\infty} \frac{1}{2\pi} \cdot e^{-\frac{1}{2} \cdot \frac{v_2 \sigma_1^4 + \sigma_1^2 (Laborhour - (\gamma_0 + Controls'\delta_1 + a_1 Health_1))^2 + \sigma_1^2 \rho_1^2 (v_2^2 - v_2) - 2\sigma_1^2 \rho_1 v_2 (Laborhour - (\gamma_0 + Controls'\delta_1 + a_1 Health_1))}{\sigma_1^2 (\sigma_1^2 - \rho_1^2)}} dv_2$$

$$= \frac{1}{\sigma_1} \int_{-\beta_0 - Varbs'\delta_2}^{+\infty} \frac{1}{2\pi} \cdot e^{-\frac{1}{2} \cdot \frac{\sigma_1^4 (\beta_0 + Varbs'\delta_2)^2 + 2\sigma_1^2 \rho_1 (Laborhour - \gamma_0 - Controls'\delta_1 - \alpha_1 Health_1)(\beta_0 + Varbs'\delta_2) + \sigma_1^2 (Laborhour - \gamma_0 - Controls'\delta_1 - \alpha_1 Health_1)^2}{\sigma_1^2 (\sigma_1^2 - \rho_1^2)}} dv_2$$

$$= \frac{1}{\sigma_1} \int_{-\beta_0 - Varbs'\delta_2}^{+\infty} \frac{1}{2\pi} \cdot e^{-\frac{1}{2} \cdot \frac{(Laborhour - \gamma_0 - Controls'\delta_1 - \alpha_1 Health_1)^2}{\sigma_1^2}} \cdot e^{-\frac{1}{2} \cdot \frac{\sigma_1^4 (\beta_0 + Varbs'\delta_2)^2 + 2\sigma_1^2 \rho_1 (\beta_0 + Varbs'\delta_2)(Laborhour - \gamma_0 - Controls'\delta_1 - \alpha_1 Health_1) + \rho_1^2 (Laborhour - \gamma_0 - Controls'\delta_1 - \alpha_1 Health_1)^2}{\sigma_1^2 (\sigma_1^2 - \rho_1^2)}} dv_2$$

$$= \frac{1}{\sigma_1} \phi\left(\frac{Laborhour - \gamma_0 - Controls'\delta_1 - \alpha_1 Health_1}{\sigma_1} \right) \Phi\left(\frac{\sigma_1^2 (\beta_0 + Varbs'\delta_2) + \rho_1 (Laborhour - \gamma_0 - Controls'\delta_1 - \alpha_1 Health_1)}{\sigma_1 \sqrt{\sigma_1^2 - \rho_1^2}} \right) \tag{A.8}$$

$$f(Laborhour, Health_1 = 0 | Varbs)$$

$$= \frac{1}{\sqrt{\sigma_1^2 - \rho_1^2}} \int_{-\infty}^{-\beta_0 - Varbs'\delta_2} \phi\left(\frac{Laborhour - (\gamma_0 + Controls'\delta_1 + a_1 Health_1 + \rho_1 v_2)}{\sqrt{\sigma_1^2 - \rho_1^2}} \right) \phi(v_2) dv_2$$

$$\frac{1}{\sqrt{\sigma_1^2 - \rho_1^2}} \int_{-\infty}^{-\beta_0 - Varbs'\delta_2} \frac{1}{2\pi} \cdot e^{-\frac{\left(\frac{Laborhour - (\gamma_0 + Controls'\delta_1 + a_1 Health_1 + \rho_1 v_2)}{\sqrt{\sigma_1^2 - \rho_1^2}}\right)^2}{2}} * e^{-\frac{v_2^2}{2}} dv_2$$

$$= \frac{1}{\sqrt{\sigma_1^2 - \rho_1^2}} \int_{-\infty}^{-\beta_0 - Varbs'\delta} \frac{1}{2\pi} \cdot e^{-\frac{1}{2} \cdot \frac{v_2 \sigma_1^4 + \sigma_1^2 (Laborhour - (\gamma_0 + Controls'\delta_1 + a_1 Health_1))^2 + \sigma_1^2 \rho_1^2 (v_2^2 - v_2) - 2\sigma_1^2 \rho_1 v_2 (Laborhour - (\gamma_0 + Controls'\delta_1 + a_1 Health_1))}{\sigma_1^2 (\sigma_1^2 - \rho_1^2)}} dv_2$$

$$= \frac{1}{\sigma_1} \int_{-\infty}^{-\beta_0 - Varbs'\delta_2} \frac{1}{2\pi} \cdot e^{-\frac{1}{2} \cdot \frac{(Laborhour - \gamma_0 - Controls'\delta_1 - \alpha_1 Health_1)^2}{\sigma_1^2}} \cdot [1 - e^{-\frac{1}{2} \cdot \frac{\sigma_1^4 (\beta_0 + Varbs'\delta_2)^2 + 2\sigma_1^2 \rho_1 (\beta_0 + Varbs'\delta_2)(Laborhour - \gamma_0 - Controls'\delta_1 - \alpha_1 Health_1) + \rho_1^2 (Laborhour - \gamma_0 - Controls'\delta_1 - \alpha_1 Health_1)^2}{\sigma_1^2 (\sigma_1^2 - \rho_1^2)}}] dv_2$$

$$= \frac{1}{\sigma_1} \phi\left(\frac{Laborhour - \gamma_0 - Controls'\delta_1 - \alpha_1 Health_1}{\sigma_1} \right) \left[1 - \Phi\left(\frac{\sigma_1^2 (\beta_0 + Varbs'\delta_2) + \rho_1 (Laborhour - \gamma_0 - Controls'\delta_1 - \alpha_1 Health_1)}{\sigma_1 \sqrt{\sigma_1^2 - \rho_1^2}} \right) \right] \tag{A.9}$$

Therefore, given *Varbs*, we derive the joint distribution of *Laborhour* and *Health₁* as follows:

$$
\begin{aligned}
& f(Laborhour, Health_1 \mid Varbs) \\
& = \left[\frac{1}{\sigma_1} \phi\left(\frac{Laborhour - \gamma_0 - Controls^{'}\delta_1 - \alpha_1 Health_1}{\sigma_1} \right) \right]^{1\{Laborhour>0\}} \\
& \cdot \left[\Phi\left(\frac{\sigma_1^2(\beta_0 + Varbs^{'}\delta_2) + \rho(Laborhour - \gamma_0 - Controls^{'}\delta_1 - \alpha_1 Health_1)}{\sigma_1\sqrt{\sigma_1^2 - \rho_1^2}} \right) \right]^{1\{Laborhour>0\}\times 1\{Health_1=0\}} \\
& \cdot \left[1 - \Phi\left(\frac{\sigma_1^2(\beta_0 + Varbs^{'}\delta_2) + \rho(Laborhour - \gamma_0 - Controls^{'}\delta_1 - \alpha_1 Health_1)}{\sigma_1\sqrt{\sigma_1^2 - \rho_1^2}} \right) \right]^{1\{Laborhour>0\}\times 1\{Health_1=1\}} \\
& \cdot \left[1 - \Phi\left(\beta_0 + Varbs^{'}\delta_2\right) - \int_{-\infty}^{-\beta_0 - Varbs^{'}\delta_2} \Phi\left(\frac{\gamma_0 + Controls^{'}\delta_1 + \alpha_1 Health_1 + \rho_1 v_2}{\sqrt{\sigma_1^2 - \rho_1^2}} \right) \phi(v_2)dv_2 \right]^{1\{Laborhour=0\}\times 1\{Health_1=0\}} \\
& \cdot \left[\Phi\left(\beta_0 + Varbs^{'}\delta_2\right) - \int_{-\beta_0 - Varbs^{'}\delta_2}^{+\infty} \Phi\left(\frac{\gamma_0 + Controls^{'}\delta_1 + \alpha_1 Health_1 + \rho_1 v_2}{\sqrt{\sigma_1^2 - \rho_1^2}} \right) \phi(v_2)dv_2 \right]^{1\{Laborhour=0\}\times 1\{Health_1=1\}}
\end{aligned}
\quad \text{(A.10)}
$$

Equation (A.10) is the same as equation (10).

A2. FIML for the ordered variable of health (Self-reported health)

When the health is an ordered variable, we have the following simultaneous equations:

$$
\begin{aligned}
&Laborhour = \max\{0, Laborhour^*\} \equiv \max\{0, \gamma_0 + Controls'\delta_1 + a_1 Health_2 + \mu_1\} \\
&Health_2^* = \chi_0 + Controls'\eta_{21} + Identify'\eta_{22} + \varepsilon_2 \equiv \chi_0 + Varbs'\eta_2 + \varepsilon_2 \\
&Health_2 = \begin{cases} 1, & -\infty < Health_2^* \le \tau_1 \\ 2, & \tau_1 < Health_2^* \le \tau_2 \\ 3, & \tau_2 < Health_2^* < +\infty \end{cases}
\end{aligned}
\tag{6}
$$

where $Cov(Varbs,\ \varepsilon_2) = 0$, $Var\begin{pmatrix}\mu_1 \\ \varepsilon_2\end{pmatrix} = \begin{pmatrix}\sigma_1^2 & \rho_2 \\ \rho_2 & 1\end{pmatrix}$, and $\mu_1 = \rho_2\varepsilon_2 + e_2$, $\rho_2 = cov(\mu_1, \varepsilon_2)$. μ_1 and ε_2 are jointly normal distribution with zero mean value.

Since we have $e_2 \sim N(0, \sigma_1^2 - \rho_2^2)$, and

$$
\begin{aligned}
Laborhour^* &= \gamma_0 + Controls'\delta_1 + a_1 Health_2 + \mu_1 \\
&= \gamma_0 + Controls'\delta_1 + a_1 Health_2 + \rho_2\varepsilon_2 + e_2
\end{aligned}
\tag{A.11}
$$

Then we arrive at:

$$
Laborhour^* |_{Varbs,\varepsilon_2} \sim N(\gamma_0 + Controls'\delta_1 + a_1 Health_2 + \rho_2\varepsilon_2, \sigma_1^2 - \rho_2^2) \tag{A.12}
$$

Following the above exercise, if $Laborhour = 0$, we have:

$$
\begin{aligned}
&P(Laborhour = 0, Health_2 = 1 | Varbs) \\
&= E\left[E\left(1\{Laborhour^* \le 0\} | \varepsilon_2, Varbs\right) | Health_2 = 1, Varbs\right] P(Health_2 = 1 | Varbs) \\
&= E\left[P\left(e_2 \le -\gamma_0 - Controls'\delta_1 - a_1 Health_2 - \rho_2\varepsilon_2 | \varepsilon_2, Varbs\right) | Health_2 = 1, Varbs\right] P(Health_2 = 1 | Varbs) \\
&= E\left[\Phi\left(-\frac{\gamma_0 + Controls'\delta_1 + a_1 Health_2 + \rho_2\varepsilon_2}{\sqrt{\sigma_1^2 - \rho_2^2}}\right) | \varepsilon_2 \le \tau_1 - \chi_0 - Varbs'\eta_2, Varbs\right] P(Health_2 = 1 | Varbs) \\
&= \int_{-\infty}^{\tau_1 - \chi_0 - Varbs'\eta_2} \Phi\left(-\frac{\gamma_0 + Controls'\delta_1 + a_1 Health_2 + \rho_2\varepsilon_2}{\sqrt{\sigma_1^2 - \rho_2^2}}\right)\phi(\varepsilon_2)d\varepsilon_2 \\
&= \Phi(\tau_1 - \chi_0 - Varbs'\eta_2) - \int_{-\infty}^{\tau_1 - \chi_0 - Varbs'\eta_2} \Phi\left(\frac{\gamma_0 + Controls'\delta_1 + a_1 Health_2 + \rho_2\varepsilon_2}{\sqrt{\sigma_1^2 - \rho_2^2}}\right)\phi(\varepsilon_2)d\varepsilon_2
\end{aligned}
\tag{A.13}
$$

$$
\begin{aligned}
&P(Laborhour=0, Health_2=2\,|\,Varbs)\\
&=E\left[E\left(1\{Laborhour^{*}\le 0\}\,|\,\varepsilon_2, Varbs\right)|\,Health_2=2, Varbs\right]P(Health_2=2\,|\,Varbs)\\
&=E\left[P\left(e_2\le -\gamma_0-Controls'\delta_1-a_1Health_2-\rho_2\varepsilon_2\,|\,\varepsilon_2, Varbs\right)|\,Health_2=2, Varbs\right]P(Health_2=2\,|\,Varbs)\\
&=E\left[\Phi\left(-\frac{\gamma_0+Controls'\delta_1+a_1Health_2+\rho_2\varepsilon_2}{\sqrt{\sigma_1^2-\rho_2^2}}\right)|\,\tau_1-\chi_0-Varbs'\eta_2<\varepsilon_2\le\tau_2-\chi_0-Varbs'\eta_2, Varbs\right]P(Health_2=2\,|\,Varbs)\\
&=\int_{\tau_1-\chi_0-Varbs'\eta_2}^{\tau_2-\chi_0-Varbs'\eta_2}\Phi\left(-\frac{\gamma_0+Controls'\delta_1+a_1Health_2+\rho_2\varepsilon_2}{\sqrt{\sigma_1^2-\rho_2^2}}\right)\phi(\varepsilon_2)d\varepsilon_2\\
&=\Phi(\tau_2-\chi_0-Varbs'\eta_2)-\Phi(\tau_1-\chi_0-Varbs'\eta_2)-\int_{\tau_1-\chi_0-Varbs'\eta_2}^{\tau_2-\chi_0-Varbs'\eta_2}\Phi\left(\frac{\gamma_0+Controls'\delta_1+a_1Health_2+\rho_2\varepsilon_2}{\sqrt{\sigma_1^2-\rho_2^2}}\right)\phi(\varepsilon_2)d\varepsilon_2
\end{aligned}
\tag{A.14}
$$

$$
\begin{aligned}
&P(Laborhour=0, Health_2=3\,|\,Varbs)\\
&=E\left[E\left(1\{Laborhour^{*}\le 0\}\,|\,\varepsilon_2, Varbs\right)|\,Health_2=3, Varbs\right]P(Health_2=3\,|\,Varbs)\\
&=E\left[P\left(e_2\le -\gamma_0-Controls'\delta_1-a_1Health_2-\rho_2\varepsilon_2\,|\,\varepsilon_2, Varbs\right)|\,Health_2=3, Varbs\right]P(Health_2=3\,|\,Varbs)\\
&=E\left[\Phi\left(-\frac{\gamma_0+Controls'\delta_1+a_1Health_2+\rho_2\varepsilon_2}{\sqrt{\sigma_1^2-\rho_2^2}}\right)|\,\varepsilon_2>\tau_2-\chi_0-Varbs'\eta_2, Varbs\right]P(Health_2=3\,|\,Varbs)\\
&=\int_{\tau_2-\chi_0-Varbs'\eta_2}^{+\infty}\Phi\left(-\frac{\gamma_0+Controls'\delta_1+a_1Health_2+\rho_2\varepsilon_2}{\sqrt{\sigma_1^2-\rho_2^2}}\right)\phi(\varepsilon_2)d\varepsilon_2\\
&=1-\Phi(\tau_2-\chi_0-Varbs'\eta_2)-\int_{\tau_2-\chi_0-Varbs'\eta_2}^{+\infty}\Phi\left(\frac{\gamma_0+Controls'\delta_1+a_1Health_2+\rho_2\varepsilon_2}{\sqrt{\sigma_1^2-\rho_2^2}}\right)\phi(\varepsilon_2)d\varepsilon_2
\end{aligned}
\tag{A.15}
$$

If *Laborhour* = *Laborhour**, for any *Laborhour>0* , we have:

$$
\begin{aligned}
&P(Laborhour, Health_2=1\,|\,Varbs)\\
&=E\left[E\left(1\{Laborhour^{*}<Laborhour\}\,|\,\varepsilon_2, Varbs\right)|\,Health_2=1, Varbs\right]P(Health_2=1\,|\,Varbs)\\
&=E\left[P\left(e_2<Laborhour-\gamma_0-Controls'\delta_1-a_1Health_2-\rho_2\varepsilon_2\,|\,\varepsilon_2, Varbs\right)|\,Health_2=1, Varbs\right]P(Health_2=1\,|\,Varbs)\\
&=E\left[\Phi\left(\frac{Laborhour-(\gamma_0+Controls'\delta_1+a_1Health_2+\rho_2\varepsilon_2)}{\sqrt{\sigma_1^2-\rho_2^2}}\right)|\,\varepsilon_2<\tau_1-\chi_0-Varbs'\eta_2, Varbs\right]P(Health_2=1\,|\,Varbs)\\
&=\int_{-\infty}^{\tau_1-\chi_0-Varbs'\eta_2}\Phi\left(\frac{Laborhour-(\gamma_0+Controls'\delta_1+a_1Health_2+\rho_2\varepsilon_2)}{\sqrt{\sigma_1^2-\rho_2^2}}\right)\phi(\varepsilon_2)d\varepsilon_2
\end{aligned}
\tag{A.16}
$$

$$P(Laborhour, Health_2 = 2 \mid Varbs)$$
$$= E\left[E\left(1\{Laborhour^* < Laborhour\} \mid \varepsilon_2, Varbs\right) \mid Health_2 = 2, Varbs \right] P(Health_2 = 2 \mid Varbs)$$
$$= E\left[P\left(e_2 < Laborhour - \gamma_0 - Controls^{'}\delta_1 - a_1 Health_2 - \rho_2\varepsilon_2 \mid \varepsilon_2, Varbs\right) \mid Health_2 = 2, Varbs \right] P(Health_2 = 2 \mid Varbs)$$
$$= E\left[\Phi\left(\frac{Laborhour - (\gamma_0 + Controls^{'}\delta_1 + a_1 Health_2 + \rho_2\varepsilon_2)}{\sqrt{\sigma_1^2 - \rho_2^2}} \right) \mid \tau_1 - \chi_0 - Varbs^{'}\eta_2 < \varepsilon_2 \le \tau_2 - \chi_0 - Varbs^{'}\eta_2, Varbs \right] P(Health_2 = 2 \mid Varbs)$$
$$= \int_{\tau_1 - \chi_0 - Varbs^{'}\eta_2}^{\tau_2 - \chi_0 - Varbs^{'}\eta_2} \Phi\left(\frac{Laborhour - (\gamma_0 + Controls^{'}\delta_1 + a_1 Health_2 + \rho_2\varepsilon_2)}{\sqrt{\sigma_1^2 - \rho_2^2}} \right) \phi(\varepsilon_2) d\varepsilon_2$$

(A.17)

$$P(Laborhour, Health_2 = 3 \mid Varbs)$$
$$= E\left[E\left(1\{Laborhour^* < Laborhour\} \mid \varepsilon_2, Varbs\right) \mid Health_2 = 3, Varbs \right] P(Health_2 = 3 \mid Varbs)$$
$$= E\left[P\left(e_2 < Laborhour - \gamma_0 - Controls^{'}\delta_1 - a_1 Health_2 - \rho_2\varepsilon_2 \mid \varepsilon_2, Varbs\right) \mid Health_2 = 3, Varbs \right] P(Health_2 = 3 \mid Varbs)$$
$$= E\left[\Phi\left(\frac{Laborhour - (\gamma_0 + Controls^{'}\delta_1 + a_1 Health_2 + \rho_2\varepsilon_2)}{\sqrt{\sigma_1^2 - \rho_2^2}} \right) \mid \varepsilon_2 > \tau_2 - \chi_0 - Varbs^{'}\eta_2, Varbs \right] P(Health_2 = 3 \mid Varbs)$$
$$= \int_{\tau_2 - \chi_0 - Varbs^{'}\eta_2}^{+\infty} \Phi\left(\frac{Laborhour - (\gamma_0 + Controls^{'}\delta_1 + a_1 Health_2 + \rho_2\varepsilon_2)}{\sqrt{\sigma_1^2 - \rho_2^2}} \right) \phi(\varepsilon_2) d\varepsilon_2$$

(A.18)

Therefore, when *Laborhour>0*, we arrive at the following equations:

$$f(Laborhour, Health_2 = 1 \mid Varbs)$$
$$= \frac{1}{\sqrt{\sigma_1^2 - \rho_2^2}} \int_{-\infty}^{\tau_1 - \chi_0 - Varbs^{'}\eta_2} \phi\left(\frac{Laborhour - (\gamma_0 + Controls^{'}\delta_1 + a_1 Health_2 + \rho_2\varepsilon_2)}{\sqrt{\sigma_1^2 - \rho_2^2}} \right) \phi(\varepsilon_2) d\varepsilon_2$$
$$= \frac{1}{\sqrt{\sigma_1^2 - \rho_2^2}} \int_{-\infty}^{\tau_1 - \chi_0 - Varbs^{'}\eta_2} \frac{1}{2\pi} \cdot e^{-\frac{1}{2}\cdot\frac{(Laborhour - \gamma_0 - Controls^{'}\delta_1 - \alpha_1 Health_2)^2 - 2(Laborhour - \gamma_0 - Controls^{'}\delta_1 - \alpha_1 Health_2)\rho_2\varepsilon_2 + \rho_2{}^2\varepsilon_2{}^2 + \varepsilon_2{}^2(\sigma_1^2 - \rho_2^2)}{\sigma_1^2 - \rho_2^2}} d\varepsilon_2$$
$$= \int_{-\infty}^{\tau_1 - \chi_0 - Varbs^{'}\eta_2} \frac{1}{2\pi} \cdot e^{-\frac{1}{2}\cdot\frac{(Laborhour - \gamma_0 - Controls^{'}\delta_1 - \alpha_1 Health_2)^2}{\sigma_1^2}} \cdot [1 - e^{-\frac{1}{2}\cdot\frac{\sigma_1{}^4(\chi_0 + Varbs^{'}\eta_2 - \tau_1)^2 + 2\sigma_1{}^2\rho_2{}^2(\chi_0 + Varbs^{'}\eta_2 - \tau_1)(Laborhour - \gamma_0 - Controls^{'}\delta_1 - \alpha_1 Health_2) + \rho_2{}^2(Laborhour - \gamma_0 - Controls^{'}\delta_1 - \alpha_1 Health_2)^2}{\sigma_1^2(\sigma_1{}^2 - \rho_2{}^2)}}] d\varepsilon_2$$
$$= \phi\left(\frac{Laborhour - \gamma_0 - Controls^{'}\delta_1 - \alpha_1 Health_1}{\sigma_1} \right)$$
$$\cdot \left[1 - \Phi\left(\frac{\sigma_1^2(\chi_0 + Varbs^{'}\eta_2 - \tau_1) + \rho_2(Laborhour - \gamma_0 - Controls^{'}\delta_1 - \alpha_1 Health_1)}{\sigma_1\sqrt{\sigma_1^2 - \rho_2^2}} \right) \right]$$

(A.19)

$$f(Laborhour, Health_2 = 2 \mid Varbs)$$

$$= \frac{1}{\sqrt{\sigma_1^2 - \rho_2^2}} \int_{\tau_1 - \chi_0 - Varbs'\eta_2}^{\tau_2 - \chi_0 - Varbs'\eta_2} \phi\left(\frac{Laborhour - (\gamma_0 + Controls'\delta_1 + a_1 Health_2 + \rho_2 \varepsilon_2)}{\sqrt{\sigma_1^2 - \rho_2^2}} \right) \phi(\varepsilon_2) d\varepsilon_2$$

$$= \frac{1}{\sqrt{\sigma_1^2 - \rho_2^2}} \int_{\tau_1 - \chi_0 - Varbs'\eta_2}^{\tau_2 - \chi_0 - Varbs'\eta_2} \frac{1}{2\pi} \cdot e^{-\frac{1}{2} \cdot \frac{(Laborhour - \gamma_0 - Controls'\delta_1 - \alpha_1 Health_2)^2 - 2(Laborhour - \gamma_0 - Controls'\delta_1 - \alpha_1 Health_2)\rho_2 \varepsilon_2 + \rho_2^2 \varepsilon_2^2 + \varepsilon_2^2 (\sigma_1^2 - \rho_2^2)}{\sigma_1^2 - \rho_2^2}} d\varepsilon_2$$

$$\frac{1}{2\pi} \cdot e^{-\frac{1}{2} \cdot \frac{(Laborhour - \gamma_0 - Controls'\delta_1 - \alpha_1 Health_2)^2}{\sigma_1^2}}$$

$$= \int_{\tau_1 - \chi_0 - Varbs'\eta_2}^{\tau_2 - \chi_0 - Varbs'\eta_2} \cdot [e^{-\frac{1}{2} \cdot \frac{\sigma_1^4 (\tau_2 - \chi_0 - Varbs'\eta_2)^2 + 2\sigma_1^2 \rho_2^2 (\tau_2 - \chi_0 - Varbs'\eta_2)(Laborhour - \gamma_0 - Controls'\delta_1 - \alpha_1 Health_2) + \rho_2^2 (Laborhour - \gamma_0 - Controls'\delta_1 - \alpha_1 Health_2)^2}{\sigma_1^2 (\sigma_1^2 - \rho_2^2)}}$$

$$- e^{-\frac{1}{2} \cdot \frac{\sigma_1^4 (\tau_1 - \chi_0 - Varbs'\eta_2)^2 + 2\sigma_1^2 \rho_2^2 (\tau_1 - \chi_0 - Varbs'\eta_2)(Laborhour - \gamma_0 - Controls'\delta_1 - \alpha_1 Health_2) + \rho_2^2 (Laborhour - \gamma_0 - Controls'\delta_1 - \alpha_1 Health_2)^2}{\sigma_1^2 (\sigma_1^2 - \rho_2^2)}}] d\varepsilon_2$$

$$= \phi\left(\frac{Laborhour - \gamma_0 - Controls'\delta_1 - \alpha_1 Health_2}{\sigma_1} \right)$$

$$\cdot \left[\begin{array}{l} \Phi\left(\frac{\sigma_1^2 (\tau_2 - \chi_0 - Varbs'\eta_2) + \rho_2 (Laborhour - \gamma_0 - Controls'\delta_1 - \alpha_1 Health_2)}{\sigma_1 \sqrt{\sigma_1^2 - \rho_2^2}} \right) \\ - \Phi\left(\frac{\sigma_1^2 (\tau_1 - \chi_0 - Varbs'\eta_2) + \rho_2 (Laborhour - \gamma_0 - Controls'\delta_1 - \alpha_1 Health_2)}{\sigma_1 \sqrt{\sigma_1^2 - \rho_2^2}} \right) \end{array} \right]$$

(A.20)

$$f(Laborhour, Health_2 = 3 \mid Varbs)$$

$$= \frac{1}{\sqrt{\sigma_1^2 - \rho_2^2}} \int_{\tau_2 - \chi_0 - Varbs'\eta_2}^{+\infty} \phi\left(\frac{Laborhour - (\gamma_0 + Controls'\delta_1 + a_1 Health_2 + \rho_2 \varepsilon_2)}{\sqrt{\sigma_1^2 - \rho_2^2}} \right) \phi(\varepsilon_2) d\varepsilon_2$$

$$= \frac{1}{\sqrt{\sigma_1^2 - \rho_2^2}} \int_{\tau_2 - \chi_0 - Varbs'\eta_2}^{+\infty} \frac{1}{2\pi} \cdot e^{-\frac{1}{2} \cdot \frac{(Laborhour - \gamma_0 - Controls'\delta_1 - \alpha_1 Health_2)^2 - 2(Laborhour - \gamma_0 - Controls'\delta_1 - \alpha_1 Health_2)\rho_2 \varepsilon_2 + \rho_2^2 \varepsilon_2^2 + \varepsilon_2^2 (\sigma_1^2 - \rho_2^2)}{\sigma_1^2 - \rho_2^2}} d\varepsilon_2$$

$$\frac{1}{2\pi} \cdot e^{-\frac{1}{2} \cdot \frac{(Laborhour - \gamma_0 - Controls'\delta_1 - \alpha_1 Health_2)^2}{\sigma_1^2}}$$

$$= \int_{\tau_2 - \chi_0 - Varbs'\eta_2}^{+\infty} \cdot [1 - e^{-\frac{1}{2} \cdot \frac{\sigma_1^4 (\tau_2 - \chi_0 - Varbs'\eta_2)^2 + 2\sigma_1^2 \rho_2^2 (\tau_2 - \chi_0 - Varbs'\eta_2)(Laborhour - \gamma_0 - Controls'\delta_1 - \alpha_1 Health_2) + \rho_2^2 (Laborhour - \gamma_0 - Controls'\delta_1 - \alpha_1 Health_2)^2}{\sigma_1^2 (\sigma_1^2 - \rho_2^2)}}] d\varepsilon_2$$

$$= \phi\left(\frac{Laborhour - \gamma_0 - Controls'\delta_1 - \alpha_1 Health_2}{\sigma_1} \right)$$

$$\cdot \left[1 - \Phi\left(\frac{\sigma_1^2 (\tau_2 - \chi_0 - Varbs'\eta_2) + \rho_2 (Laborhour - \gamma_0 - Controls'\delta_1 - \alpha_1 Health_2)}{\sigma_1 \sqrt{\sigma_1^2 - \rho_2^2}} \right) \right]$$

(A.21)

Therefore, given *Varbs*, we derive the joint distribution of *Laborhour* and *Health₁* as follows:

$$
\begin{aligned}
& f(Laborhour, Health_2 \mid Varbs) \\
& = \left[\phi\left(\frac{Laborhour - \gamma_0 - Controls'\delta_1 - \alpha_1 Health_2}{\sigma_1} \right) \right]^{1\{Laborhour>0\}} \\
& \cdot \left[1 - \Phi\left(\frac{\sigma_1^2(\chi_0 + Varbs'\eta_2 - \tau_1) + \rho(Laborhour - \gamma_0 - Controls'\delta_1 - \alpha_1 Health_2)}{\sigma_1\sqrt{\sigma_1^2 - \rho_2^2}} \right) \right]^{1\{Laborhour>0\}\times 1\{Health_2=1\}} \\
& \cdot \left[\begin{array}{l} \Phi\left(\dfrac{\sigma_1^2(\tau_2 - \chi_0 - Varbs'\eta_2) + \rho(Laborhour - \gamma_0 - Controls'\delta_1 - \alpha_1 Health_2)}{\sigma_1\sqrt{\sigma_1^2 - \rho_2^2}} \right) \\ -\Phi\left(\dfrac{\sigma_1^2(\tau_1 - \chi_0 - Varbs'\eta_2) + \rho(Laborhour - \gamma_0 - Controls'\delta_1 - \alpha_1 Health_2)}{\sigma_1\sqrt{\sigma_1^2 - \rho_2^2}} \right) \end{array} \right]^{1\{Laborhour>0\}\times 1\{Health_2=2\}} \\
& \cdot \left[1 - \Phi\left(\frac{\sigma_1^2(\tau_2 - \chi_0 - Varbs'\eta_2) + \rho(Laborhour - \gamma_0 - Controls'\delta_1 - \alpha_1 Health_2)}{\sigma_1\sqrt{\sigma_1^2 - \rho_2^2}} \right) \right]^{1\{Laborhour>0\}\times 1\{Health_2=3\}} \\
& \cdot \left[\Phi(\tau_1 - \chi_0 - Varbs'\eta_2) - \int_{-\infty}^{\tau_1 - \chi_0 - Varbs'\eta_2} \Phi\left(\frac{\gamma_0 + Controls'\delta_1 + \alpha_1 Health_2 + \rho_2\varepsilon_2}{\sqrt{\sigma_1^2 - \rho_2^2}} \right) \phi(\varepsilon_2) d\varepsilon_2 \right]^{1\{Laborhour=0\}\times 1\{Health_2=1\}} \\
& \cdot \left[\Phi(\tau_2 - \chi_0 - Varbs'\eta_2) - \Phi(\tau_1 - \chi_0 - Varbs'\eta_2) - \int_{\tau_1 - \chi_0 - Varbs'\eta_2}^{\tau_2 - \chi_0 - Varbs'\eta_2} \Phi\left(\frac{\gamma_0 + Controls'\delta_1 + \alpha_1 Health_2 + \rho_2\varepsilon_2}{\sqrt{\sigma_1^2 - \rho_2^2}} \right) \phi(\varepsilon_2) d\varepsilon_2 \right]^{1\{Laborhour=0\}\times 1\{Health_2=2\}} \\
& \cdot \left[1 - \Phi(\tau_2 - \chi_0 - Varbs'\eta_2) - \int_{\tau_2 - \chi_0 - Varbs'\eta_2}^{+\infty} \Phi\left(\frac{\gamma_0 + Controls'\delta_1 + \alpha_1 Health_2 + \rho_2\varepsilon_2}{\sqrt{\sigma_1^2 - \rho_2^2}} \right) \phi(\varepsilon_2) d\varepsilon_2 \right]^{1\{Laborhour=0\}\times 1\{Health_2=3\}}
\end{aligned}
$$

(A.22)

Equation (A.22) is the same as equation (11).
